# *Leishmania donovani* persistence and circulation causing cutaneous leishmaniasis in unusual-foci of Nepal

**DOI:** 10.1038/s41598-023-37458-6

**Published:** 2023-07-29

**Authors:** Tinmaya Rai, Srijan Shrestha, Sabita Prajapati, Anup Bastola, Niraj Parajuli, Pragya Gautam Ghimire, Parmananda Bhandari, Kishor Pandey, Manju Jain, Greg Matlashewski, Rachel Bras-Goncalves, Krishna Das Manandhar

**Affiliations:** 1 Central Department of Biotechnology, Tribhuvan University, Kirtipur, Kathmandu, 44600 Nepal; 2 Sukraraj Tropical and Infectious Disease Hospital, Kathmandu, 44600 Nepal; 3 Bir Hospital, Kathmandu, 44600 Nepal; 4 Nepalganj Medical College and Teaching Hospital, Nepalganj, 21900 Nepal; 5 Central Department of Zoology, Tribhuvan University, Kirtipur, Kathmandu, 44600 Nepal; 6 Department of Biochemistry, Central University of Punjab, Bathinda, Punjab 151401 India; 7 Department of Microbiology and Immunology, McGill University, Montreal, QC H3A 0G4 Canada; 8 INTERTRYP, University Montpellier, IRD, CIRAD, 34000 Montpellier, France

**Keywords:** Infectious diseases, Phylogenomics, Parasite genetics

## Abstract

Cutaneous leishmaniasis cases have increased dramatically in recent years in Nepal. The study offers molecular identification of the *Leishmania* species using 40 patient’s aspiration biopsy samples, targeting markers kinetoplast minicircle DNA (kDNA) and internal transcribed spacer-1 (ITS1). Among molecularly diagnosed 22 cutaneous leishmaniasis cases, *L. donovani* complex was identified in 13 instances and *L. major* in 9 cases. The ITS1 PCR was positive in 12 of the positive nested- kDNA PCR cases (12/22), confirming *L. donovani* complex in seven of the cases and *L. major* in five of the cases. In addition, the study conclude that concurrent occurrence of atypical cutaneous infections caused by *L. donovani* parasite in 59.1% of cases and typical cutaneous infections caused by *L. major* parasite in 40.9% of cases. A Phylogentic analaysis showed that the detected *L. donovani* species present null genetic distances from seven references of *L. donovani*, but slight differences between ITS1 sequences and not grouped into a significant monophyletic cluster.

## Introduction

Leishmaniasis is a vector-borne disease caused by obligate intracellular protozoan parasite, *Leishmania* with more than twenty pathogenic species that infect human. It is a disease of the poor society, occurring mostly in remote rural villages with poor housing and little or no access to modern health-care facilities^1^. Leishmaniasis has been a severe health problem in tropical and subtropical regions with over 1 billion people at risk of infection living in 98 endemic countries in 5 continents^2,3^. It includes the spectrum of disease from self-healing skin disease to diffuse cutaneous ailment, mucocutaneous disease and visceral leishmaniasis, the fatal one if not treated^4^. Cutaneous leishmaniasis is more widely distributed with about one-third of cases occurring in each of three epidemiological regions, the Americas, the Mediterranean basin, and western Asia from the Middle East to Central Asia^5^.

Clinical symptoms are used to make a preliminary diagnosis of cutaneous leishmaniasis. The parasitological diagnosis, which is based on direct identification of amastigotes in microscopy smears from infected tissues, is still the gold standard. Although the procedure is quite specific, it is insufficiently sensitive and need skilled technicians^6,7^. Furthermore, the culture protocol has chances of contaminations and is time consuming^8^. The molecular tools based on a variety of molecular markers and polymerase chain reaction (PCR) have been developed. PCR-based protocols have high speed and sensitivity of species-specific leishmaniasis diagnosis compared to the conventional techniques such as microscopy and parasite culture^9^.

Nepal, a South Asian Country, is in the very interesting geographical location of the world having the plain tropical in the South to the highest Mount Everest Himalaya in the North. Visceral leishmaniasis is endemic in Nepal since 1950s, primarily in the southeastern region of the Terai plain, along India^10,11^. Regarding the cutaneous leishmaniasis, Parija et al.^10^ reported the first case as imported in 1998. In 2006, a patient from Nepal visited to Safdarjung Hospital of New Delhi (India) for the treatment of skin disease was initially suspected as a case of basal cell carcinoma, but with skin clinical manifestations similar to a cutaneous leishmaniasis, a molecular diagnostic test (kDNA PCR) made it possible to diagnose cutaneous leishmaniasis caused by *Leishmania (L.) major* followed by the most appropriate treatment^12^. Increasing trends of cutaneous leishmaniasis have been reported in different parts of Nepal^11,13^. From 2016 to 2019, Pandey et al. reported 42 cutaneous leishmaniasis cases referring to Epidemiology and Diseases Control Division (EDCD), Ministry of Health and Population, Nepal as diagnosed on the basis of clinical presentation and laboratory findings^11^. Among these data, a very clear increment was observed between 2017 (2 cases) and 2018 (18 cases), and 21 new cutaneous leishmaniasis cases were reported in 2019 alone^11^. Biological diagnosis of cutaneous leishmaniasis is based on microscopic examination by fine needle aspiration cytology^13^, histopathology^14^ and serological rK39 diagnostic tests^11^. The first PCR-based molecular diagnosis to diagnose leishmaniasis in Nepal was performed in 2017 by Prof. Manandhar’s Infectious and Viral Disease Research Laboratory of the Central Department of Biotechnology, Tribhuvan University, Kirtipur, Kathmandu, Nepal^15^. Since then molecular diagnosis is carefully performed in the suspected cases of cutaneous leishmaniasis. Thus, a large number of cases have been confirmed by PCR-based diagnosis with identification of the *Leishmania* species involved. Patients came from foci known to be endemic for visceral leishmaniasis as well as previously non-endemic areas of Nepal. In addition, the sequences of the CL isolated strains provided new evidence for the circulating species including *L. donovani* with dermotropic cutaneous manifestation in Nepal.

## Results

### Demographic and geographic distribution of cutaneous leishmaniasis cases

**Figure 1:**
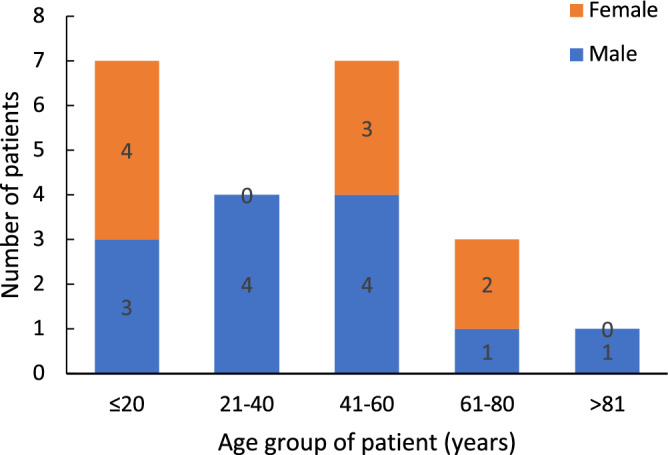
Age & gender wise distribution of cutaneous leishmaniasis positive cases.

In this study, interestingly a good numbers of suspected cases (n = 40) visited the hospitals on their own initiative in two-year study period. Among them, 65% (n = 26) were males and 35% (n = 14) were females. Among 22 (55%) cutaneous leishmaniasis cases as confirmed by this study using parasitological and/or molecular diagnosis, genderwise disribution showed 59.09% (n = 13) male while 40.9% (n = 9) were female. The research data showed that highest (n = 7) number positive cutaneous leishmaniasis cases fell into age group ≤ 20 years and in 41–60 years age group while the lowest (n = 1) number fell into age groups > 80 years (Fig. 1).

**Figure 2:**
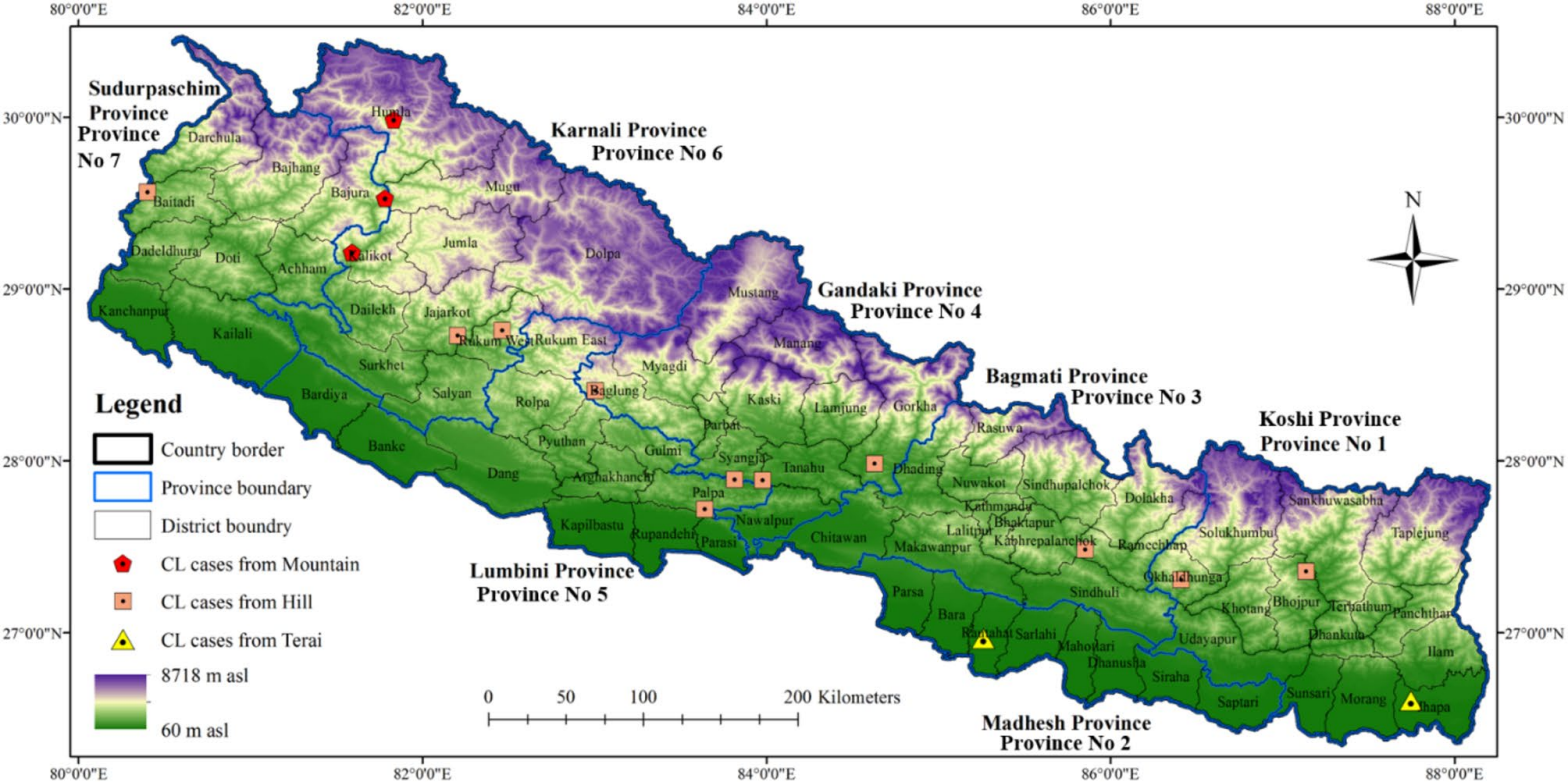
Map showing the distribution of positive cases of cutaneous leishmaniasis (CL) in the geographic regions, districts and provinces of Nepal. Map was prepared using QGIS 3.30 (http://qgis.osgeo.org).

Cutaneous leishmaniasis cases were detected from all the geographical landscapes of Terai (latitude 26°), hill (latitude 27°–29°) and mountain (latitude 29°) regions. The coverage of cutaneous leishmaniasis cases was seen in all provinces encompassing 16 districts during the period of this study (Table 1, Fig. 2). The highest number of positive cutaneous leishmaniasis cases were found in hill (n = 13) followed by mountain (n = 7) and least in the Terai (n = 2) region covering 11, 3 and 2 districts of Nepal respectively. Similarly, Province Gandaki (Province 4), Province Karnali (Province 6) and Province Sudur Pashchim (Province 7) had equal five cutaneous leishmaniasis cases. Among the two districts recorded with prevalence in Province 7, Bajura district had three cases and Baitadi district had two cases. In Province 4; Gorkha, Tanahun, Baglung and Syangja districts had total five positive cases. In province 6, three cases were from the Kalikot district, and two other cases were from Jajarkot and Humla districts. There were two cases in Province Lumbini (Province 5) from Palpa and Rukum districts. In Province Koshi (Province 1), three cases were from Jhapa, Bhojpur and Okhaldhunga districts and only one case in each Province Bagmati (Province 3) and Province Madhesh (Province 2) from Ramechhap and Rautahat districts respectively (Table 1). In reference to national and international travel history of the patients, only five patients reported their travel to different countries, namely Saudi Arabia, United Arab Emirates (Dubai), India and UK, while only five patients reported their travel to another district or province than his place of residence (Supplementary Table S-II).

**Table 1:** Distribution of cutaneous leishmaniasis cases according to geographic regions, province, and districts with travel history.

Geo graphic region	Province number (Name)	District	VL Endemicity	GPS coordinates of district’s center**		CL cases ***	General information of patient		CL etiology		National VL elimina tion lunched district
				Latitude	Longitude		Age (year)	Gender	*L. major*	*L. donovani*	
Hill	1(Koshi)	Bhojpur	Endemic	27°21′32.868′′	87°8′5.136′′	1	43	M		CL27	Yes
		Okhal dgunga	Endemic	27°18′35.748′′	86°24′31.608′′	1	58	M	CL29		Yes
	3 (Bagmati)	Rame chhap	Doubtful endemic	27°29′11.868′′	85°51′6.912′′	1	15	M	CL12		No
	4 (Gandaki)	Tanahun	Doubtful endemic	27°53′18.348′′	83°58′28.992′′	1	85	M	CL8		No
		Syangja	Doubtful endemic	27°53′32.82′′	83°48′45.612′′	1	67	F		CL10	No
		Gorkha*	Doubtful endemic	27°59′9.276′′	84°37′34.392′′	2	54	F	CL17		No
							66	F	CL23		No
		Baglung	Non-endemic	28°24′36.648′′	83°0′7.956′′	1	48	M	CL36		No
	5 (Lumbini)	Palpa	Endemic	27°43′13.548′′	83°38′21.66′′	1	23	M		CL15	Yes
		Rukum*	Doubtful endemic	28°45′39.312′′	82°27′39.456′′	1	20	M		CL35	No
	6 (Karnali)	Jajarkot*	Doubtful endemic	28°43′51.6′′	82°12′10.26′′	1	35	M		CL22	No
	7 (Sudur Pashchim)	Baitadi	Doubtful endemic	29°33′54.072′′	80°23′55.752′′	2	54	F		CL14	No
							60	F		CL25	No
Mountain	6 (Karnali)	Humla	Doubtful endemic	29°59′12.192′′	81°49′59.34′′	1	33	M		CL16	No
		Kalikot*	Doubtful endemic	29°12′49.896′′	81°35′23.532′′	3	19	F		CL2	No
							9	F	CL5		No
							61	M		CL32	No
	7 (Sudur Pashchim)	Bajura	Doubtful endemic	29°31′47.568′′	81°46′59.268′′	3	16	F		CL18	No
							15	F		CL19	No
							11	M		CL40	No
Terai	1(Koshi)	Jhapa	Endemic	26°36′25.308′′	87°44′41.748′′	1	55	M	CL39		Yes
	2 (Madhesh Pradesh)	Rautahut	Endemic	26°58′9.156′′	85°15′37.296′′	1	26	M	CL13		Yes

*District spread with hilly and mountainous areas.

**GPS coordinates the center of the districts.

***Number of cases with positive parasitological and molecular diagnosis or only molecular diagnosis. Male (M); Female (F); NA: Not Available.

### Clinical manifestations of the cutaneous lesions

**Figure 3:**
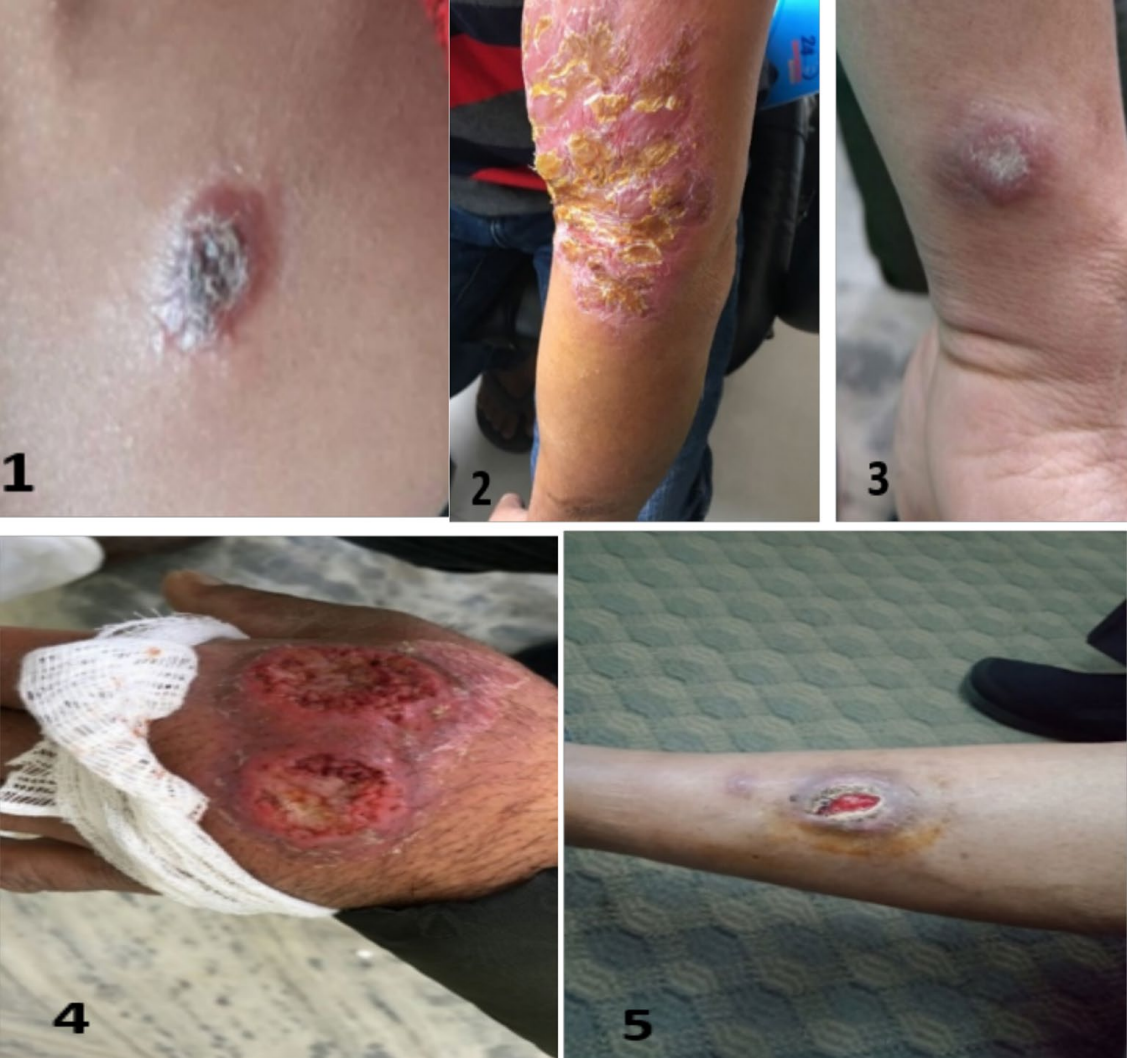
Clinical features of cutaneous leishmaniasis, (1) Dry crusted lesion in upper chest (2) Dry erythematic plaque in arm, (3) Dry and raised lesion in hand, (4) Central invaginated and moist in hand, (5) Ulcerated lesion with Central invagination on left leg.

In the patients visited to hospital with leishmaniasis suspicion, 65% (26/40) lesions were dry type, while 35% (14/40) were moist with central invaginations. Within the dry type, 46.15% (12/26) lesions had scales or crusted, 19.2% (5/26) had bulged appearance or nodulated and remaining had swollen erythematic appearance (Table 2). Most of the lesions 77.5% (31/40) were localized while 22.5% (9/40) were dispersed lesions. Similarly, most of the lesions 60% (24/40) had single lesion while 40% (16/40) had multiple lesions. The lesions in the suspected cases were in the different exposed part of the body, mostly in the face (n = 19, 47.5%), followed by hand (n = 5, 12.5%), neck (n = 5, 12.5%), leg (n = 3, 7.5%), and abdominal part (n = 3, 7.5%) (Table 2). Others having multiple lesions on these parts in the five patients (n = 5, 12.5%) (Supplementary Table S-III). In the patients with confirmed cutaneous leishmaniasis, the clinical manifestations observed were lesions with different characteristics of dry and moist type (Fig. 3, Supplementary Table S-III). Uncovered front body areas of skin were the most affected like the face, nose, lip, chin, neck and chest in 11 cutaneous leishmaniasis cases (50% of cases) while the limb lesions were noted in 6 patients (27.2%), [hand (n = 3, 13.6%) and leg/thigh (n = 3, 22.7 13.6%)] (Table 2). In four cases of cutaneous leishmaniasis the lesions were distributed on more than one site of the body (18.2%). One case of cutaneous leishmaniasis was found in which the lesion was on the waist (4.5%) (Supplementary Table S-III). Among the confirmed cases of cutaneous leishmaniasis, one had the history of visceral leishmaniasis and rest of the 21 cases had no history of visceral leishmaniasis. The case had visceral leishmaniasis 16 years ago from the date of recent visit to the hospital with complain of dry crusted erythematic lesion on the face covering nose and mouth for last 4 years. The causative species was detected as *L. donovani* according to the kDNA PCR result (720 bp). Out of the 22 positive patients diagnosed, 10 were already on medicines such as antifungal, antibiotics, antileishmaniasis and antituberculosis.

**Table 2:** Clinical diagnosis and related information to visceral leishmaniasis of cutaneous leishmaniasis cases.

Patient’s ID	Clinical manifestations of the cutaneous lesions				Previous clinical history of VL			Family and neighbors
	No. of lesions	Type of lesions	Leisons appearance	Location of lesions	VL (yes or No)	PKDL after a VL	PKDL without previous VL	VL or PKDL history of someone close
CL27	1	Dry	Erythematic	Face	Yes	No	No	No
CL29	1	Dry	Erythematic	Hand	No	No	No	No
CL12	1	Dry	Crusted	Leg	No	No	No	No
CL8	1	Moist	Central depression	Hand	No	No	No	No
CL10	1	Dry	Papular	Neck	No	No	No	No
CL17	1	Moist	Central depression	Thigh	No	No	No	No
CL23	Multiple	Dry	Crusted	Waist	No	No	No	No
CL36	Multiple	Moist	Central depression	Hand and leg	No	No	No	No
CL15	1	Moist	Ulcerated	Neck	No	No	No	No
CL35	3	Dry	Nodulated	Hand	No	No	No	No
CL22	3	Moist	Ulcerated	Hand, Nose	No	No	No	No
CL14	3	Dry	Crusted	Face	No	No	No	No
CL25	2	Moist	Central depression	Lip, Hand	No	No	No	No
CL16	1	Moist	Central depression	Lip	No	No	No	No
CL2	1	Dry	Nodulated	Face	No	No	No	No
CL5	1	Dry	Bulged	Chin	No	No	No	No
CL32	1	Moist	Ulcerated	Face	No	No	No	No
CL18	1	Dry	Crusted	Chest	No	No	No	No
CL19	1	Dry	Crusted	Lip	No	No	No	No
CL40	2	Dry	Crusted	Face	No	No	No	No
CL39	3	Moist	Central depression	Leg	No	No	No	No
CL13	Multiple	Dry	Crusted	Hand, leg and Lip	No	No	No	No

*VL* visceral leishmaniasis, *PKDL* post-kala-azar dermal leishmaniasis.

### Observation of amastigote in smear and promastigote in culture

Among 40 cutaneous leishmaniasis suspected cases, LD bodies stained with Giemsa were observed only in twelve cases (Table 3). The infected tissue inoculated culture flask had growth of flagellated promastigote (Supplementary Fig. S-I) confirming the infection by *Leishmania spp.* parasites after 7 days in one case. The percentage of detection were 54.5.5% and 4.5% respectively for amastigotes and promastigotes.

**Table 3:** Parasitological and molecular analyses (PCR, RFLP and sequencing) of cutaneous leishmaniasis patients.

Patient’s ID	Biological diagnosis						Analysis of ITS1 PCR products		
	Parasitological diagnosis	kDNA nested-PCR (n = 22) [Amplicon size (bp)]			ITS-1 PCR (n = 12) [Amplicon size (bp)]		RFLP (band pattern)	NCBI Genbank ID and sequence length	Species confirmation
	Microscopic observation	PCR-1	PCR-2	Species identified	Amplicon size (bp)	Species confirmed	(n = 12)		
CL27	Not Detected	NB	~ 720	*L. donovani*	ND	ND	ND	NA	
CL29	Amastigotes&Promastigotes	NB	~ 600	*L. major*	~ 340 bp	*L. major*	210 bp, 180 bp	ON146455 (337 bp)	*L. major*
CL12	Amastigotes	~ 700 bp	~ 600	*L. major*	~ 340 bp	*L. major*	210 bp, 180 bp	NA	*L. major*
CL8	Amastigotes	~ 700 bp	~ 600	*L. major*	ND	ND	ND	NA	
CL10	Not detected	NB	~ 720	*L. donovani*	ND	ND	ND	NA	
CL17	Amastigotes	~ 700 bp	~ 600	*L. major*	~ 340 bp	*L. major*	210 bp, 180 bp	ON146453 (337 bp)	*L. major*
CL23	Amastigotes	NB	~ 600	*L. major*	~ 340 bp	*L. major*	210 bp, 180 bp	ON146454 (337 bp)	*L. major*
CL36	Not detected	NB	~ 600	*L. major*	~ 340 bp	*L. major*	210 bp, 180 bp	NA	*L. major*
CL15	Amastigotes	NB	~ 720	*L. donovani*	ND	ND	ND	NA	
CL35	Not detected	NB	~ 720	*L. donovani*	ND	ND	ND	NA	
CL22	Amastigotes	NB	~ 720	*L. donovani*	~ 320 bp	*L. donovani*	180 bp, 75 bp, 50 bp	ON146458 (321 bp)	*L. donovani*
CL14	Not detected	NB	~ 720	*L. donovani*	ND	ND	ND	NA	
CL25	Not detected	NB	~ 720	*L. donovani*	~ 320 bp	*L. donovani*	180 bp, 75 bp, 50 bp	ON146459 (321 bp)	*L. donovani*
CL16	Not detected	NB	~ 720	*L. donovani*	~ 320 bp	*L. donovani*	180 bp, 75 bp, 50 bp	NA	*L. donovani*
CL2	Amastigotes	~ 800 bp	~ 720	*L. donovani*	~ 320 bp	*L. donovani*	180 bp, 75 bp, 50 bp	NA	*L. donovani*
CL5	Amastigotes	NB	~ 600	*L. major*	ND	ND	ND	NA	
CL32	Amastigotes	NB	~ 720	*L. donovani*	~ 320 bp	*L. donovani*	180 bp, 75 bp, 50 bp	NA	*L. donovani*
CL18	Amastigotes	NB	~ 720	*L. donovani*	~ 320 bp	*L. donovani*	180 bp, 75 bp, 50 bp	ON146457 (321 bp)	*L. donovani*
CL19	Not detected	NB	~ 720	*L. donovani*	ND	ND	ND	NA	
CL40	Not detected	NB	~ 720	*L. donovani*	~ 320 bp	*L. donovani*	180 bp, 75 bp, 50 bp	ON146460 (321 bp)	*L. donovani*
CL39	Not detected	NB	~ 600 bp	*L. major*	ND	ND	ND	NA	
CL13	Amastigotes	NB	~ 600 bp	*L. major*	ND	ND	ND	NA	

*NB* no band observed on agarose gel, *ND* not determined, *NA* not available.

### Molecular diagnosis of cutaneous leishmaniasis and identification of causative species by minicircle kDNA PCR

**Figure 4:**
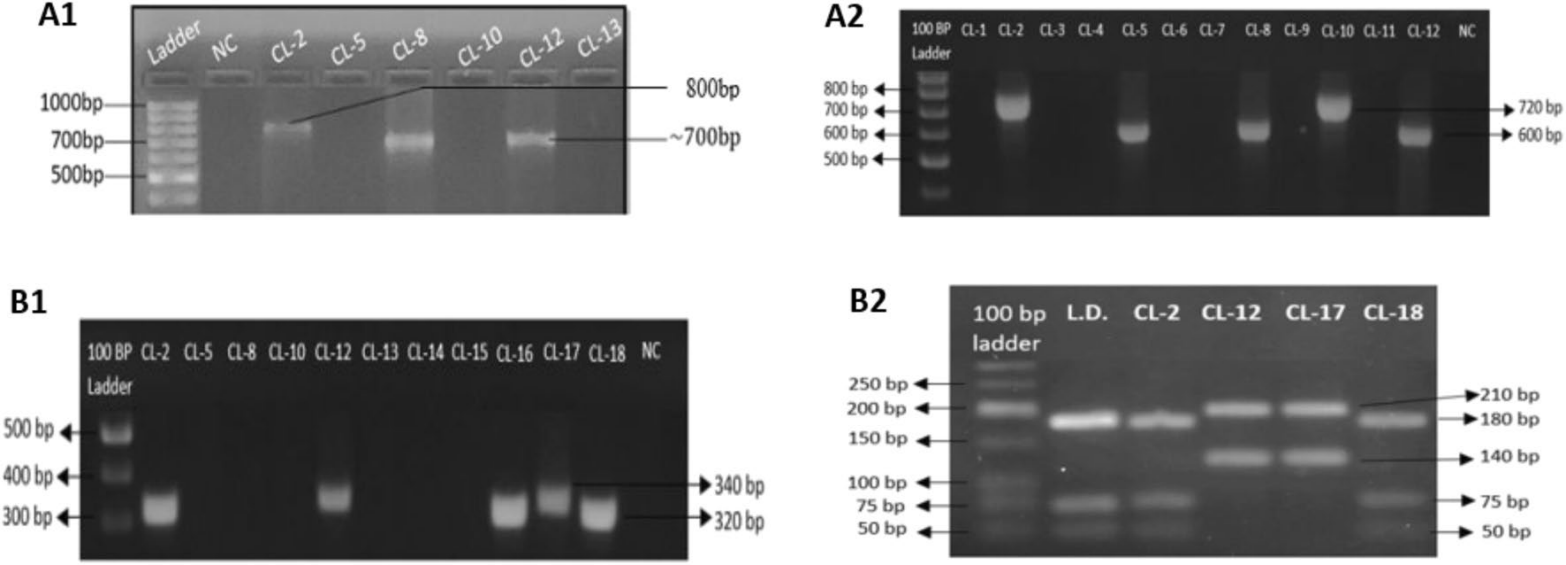
Agarose gel electrophoresis visualization of kDNA, ITS1 and ITS1-RFLP. (A1) Minicircle kDNA PCR-1 assay of clinical samples. (A2) Minicircle kDNA PCR-2 assay of clinical samples. PCR 1 amplicons of DNA isolated from the skin biopsies run for gel electrophoresis on 1.5% agarose gel and analysis. B1) ITS1 PCR assay of clinical samples. DNA isolated from the skin biopsies from the lesion was amplified and analyzed on 1.5% agarose gel. B2) ITS1 RFLP assay of ITS1 PCR positive samples. The grouping of the gels cropped were from different gels. The full length gels are included in supplementary information file. The size of the bands are defined as indicated by the arrows. Ladders-100 bp DNA ladder (Solis Biodyne), NC-Negative Control, L.D.- Control of L. donovani (LEM 138), CL- Cutaneous leishmaniasis sample.

The nested minicircle kDNA PCR, run along with negative control, detected 55% (22/40 suspected cases) to be PCR-positive cutaneous leishmaniasis cases. The amplicon sizes ranged from 700 to 800 bp in PCR-1 products whenever band was seen and the two bands observed in PCR-2 (Fig. 4A1,A2, Table 3) confirmed the cutaneous leishmaniasis in the suspected cases. The two bands of PCR-2 indicated different *Leishmania* species circulating among the study population. Further, the amplicons of 600 bp and 720 bp confirmed etiologic species of *L. major* and *L. donovani*, respectively. There were 9 cases of cutaneous leishmaniasis caused due to the species *L.major* and 13 cases were due to *L. donovani.*

### Confirmative identification of Leishmania species by ITS1 PCR and RFLP

PCR using specific primers for ITS1 gene amplified 12 samples (54.5%) out of 22 minicircle kDNA PCR positive samples. The two distinct amplicons were of about 320 bp (*L donovani*) and 340 bp (*L major*) in seven and five samples respectively (Fig. 4B1, Table 3) validating the species identification results of kDNA in which the ITS1 gene amplicons were expressed. Further ahead, the restriction digestion of ITS1 amplicons by endonuclease *Hae III* again generated two types of band patterns. The samples showing 340 bp amplicon in ITS1 PCR showed a digestion pattern with bands of around 210 bp and 140 bp amplicons while the restriction enzyme digestion of 320 bp amplicon showed a pattern with three bands of around 180, 75 and 50 bp (Fig. 4B2, Table 3). The later pattern showed similarity with that of the reference strain LEM 138 *L. donovani* parasite (provided by Prof. Shyam Sundar, IMS, BHU, India) causing visceral leishmaniasis. Whereas the two band patterns 210 and 140 bp is similar with that of the *Leishmania major*^12^. This analysis was confirmed again for 12 samples with outcome of *L. donovani* (n = 7) and *L. major* (n = 5) as the two cutaneous leishmaniasis causing parasite species are circulating in Nepal.

### Evolutionary linkage based on sequencing and phylogenetic analysis

The sequence of ITS1 region amplified by using LITSR-L5.8S primers of seven tissue samples (CL-17, CL-18, CL-22, CL-23, CL-25, CL-29 and CL-40) and one from the culture (CL-29) showed two different compositions of base pairs (Table 3). The length of the sequences from 4 samples (CL17, CL23, CL29Tissue, CL29Culture) were of 337 bp (301 bp without primers) that of *L. major*, while rest of the 4 samples (CL18, CL22, CL25 and CL40) showed of 321 bp (285 bp without primers) that of *L. donovani*. NCBI BLAST analysis of these sequences revealed 99.12% and 99.47% homology with corresponding *L. major* (GenBank Accession Number: MT023531.1) and *L. donovani* (GenBank Accession Number: MW053328.1) sequences, respectively.

**Figure 5:**
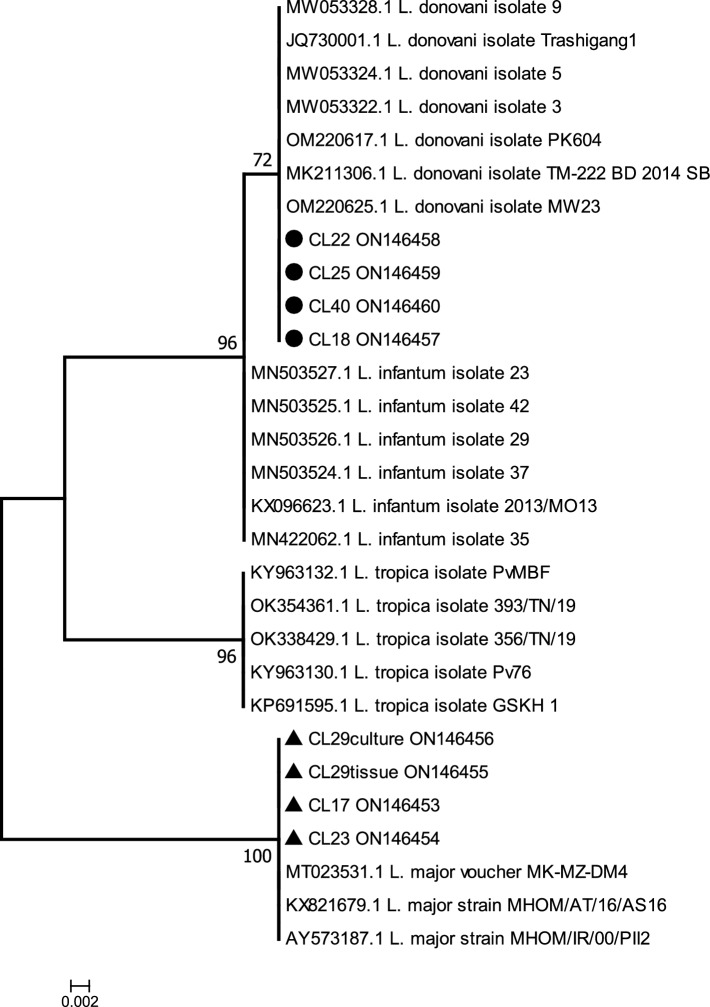
Maximum Likelihood (ML) tree of ITS1 sequences from *Leishmania* species. ML tree of 29 ITS1 sequences from four *Leishmania* species, constructed by the software IQ-TREE v.2.2.0 from an alignment of 312 nucleotide positions. This tree showing the highest log likelihood (− 554.785) is based on the best model (K2P) determined by the program itself. The percentages of bootstrap values used as phylogenetic test and based on 50,000 replications are shown at the tree nodes. The sequences are labelled with their accession numbers and isolate names. The sequences CL17, CL23, CL29Culture and CL29Tissue, belonging to L. major and identified by black triangle symbols, and CL18, CL22, CL25 and CL-40 belonging to L. donovani and identified by black circle symbols are our isolates from Nepal of years 2018–2019.

The ML tree constructed by IQ-TREE (Fig. 5) clearly confirmed that the samples CL17, CL23, CL29_Culture and CL29_Tissue belong to *L. major* species evidenced by a null genetic distance from three references of *L. major* all clustered into the same significant monophyletic clade (bootstrap value of 100%)*.* The sequences were slightly different from the *L. major* references where gaps appeared on the sequence alignment at position 17, 57 and 58 (Supplementary data S-IV). In the same way, the ML tree confirmed that the other four samples (CL18, CL22, CL25 and CL40) do belong to *L. donovani* species as their genetic distances from seven references of *L. donovani* were null. Here too, some slight differences between sequences appeared by presence/absence of gaps at positions 17, 52, 55 and 56 on the sequence alignment (Supplementary data S-IV).

It has been noted that the *L. donovani* sequences were not grouped into a significant monophyletic cluster (only 72% bootstrap value) whereas the *L. donovani* plus *L. infantum* cluster was significantly monophyletic (96% bootstrap value). This indicates that the ITS1 marker is probably not sufficient to separate these extremely closely related species.

## Discussion

In recent years, cutaneous leishmaniasis has emerged as an indigenous disease in Nepal, with increasing cases every year^11^. Moreover, lack of specialized knowledge causes medical practitioners to misdiagnose cases of cutaneous leishmaniasis as lupus vulgaris (well-defined skin-colored to erythematous plaque)^14,15^, basal cell carcinoma (nodular or nodulo-ulcerative lesions)^12^ and leprosy, which is characterized by discolored (pale or reddish) and numb patch of skin^16^. So, the patients who visited the hospitals with complaints were previously treated without a confirmed diagnosis for leishmaniasis or other skin conditions. It has been noted that some drugs are effective for treating *L. major*^17,18^, which may have hide the leishmaniasis cases. Cutaneous lesions can either be a single, limited skin lesion or multiple, large, locally destructive skin lesions.

Leishmaniasis is diagnosed in many endemic areas based on clinical symptoms supported by a diagnostic test, such as the rK39 fast diagnostic test, and microscopic amastigotes observation, which requires a highly qualified technical individual. However, the reference microscopic diagnostic has a limited sensitivity and frequently returns false negative results^19^. The amplification of the parasite’s kDNA gene has been reported as the most sensitive diagnostic test (98.7%) for the diagnosis of leishmaniasis^6,20^. However Nepal did not use PCR as a diagnostic tool until very recently that why the causative protozoan parasites of Nepalese cutaneous leishmaniasis cases remained unknown till 2018 before Prof. Manandhar’s group detected *L. major* and *L. donovani* for the first time using PCR-based molecular diagnosis and species identification^15^.

Species identification was the major aim of the present study to evaluate and identify the *L. donovani* persistence causing cutaneous leishmaniasis in Nepalese context, which have been done by PCR, restriction fragment length polymorphism (RFLP) and sequencing. The study made it possible to carry out minicircle kDNA based nested PCR test as the confirmatory diagnostic tool in Nepal for medically suspected microscopically negative cases. In this study, from skin lesion fine-needle aspirations, *Leishmania* kDNA PCR detected 22 positive cases (55%) out of 40 suspected patients being able to sort out symptomatically similar non-leishmaniasis cases. All the tools were able to discriminate *Leishmania* species circulating in Nepal. The 22 kDNA PCR positive samples were identified either as *L. donovani* (n = 13) or *L major* (n = 9). It showed that atypical *L. donovani* parasites was dominant with 59.1% to cause cutaneous leishmaniasis in Nepal over the typical etiological agent as *L. major*. The diagnostic and species identification tests were reconfirmed by ITS1 PCR which could detect 54.5% (n = 12/22) only and among them 5 cases as *L. major* out of 9 confirmed positive by kDNA PCR skipping the other 4 as undetected. Similarly, only the 7 cases of *L donovani* could be confirmed by ITS1 PCR among the 13 detected by kDNA (Table 3). The sensitivity of the kDNA PCR is higher than that of ITS1 PCR as the copy number of the kDNA target is about 10,000 copies per cell^20^ which is higher than ITS1 target regions having approximately 20–400 copies only^21^. So, kDNA PCR can detect DNA equivalent to < 0.001 parasites per reaction while the ITS1 PCR detects DNA equivalent to 0.2 parasites per reaction^6^. During the present study, parasitological diagnosis only confirmed twelve cases of cutaneous leishmaniasis similar to molecular diagnosis by ITS1 PCR among the 22 kDNA PCR positive cases. As already described by Bensoussan et al.^6^, diagnosis by kDNA PCR shows a higher sensitivity compared to parasitological diagnosis or ITS1 PCR. Therefore, the present study recommends kDNA PCR as an additional diagnostic technique, as also suggested by Pezeshkpoor et al.^22^.

The species identification were further confirmed by RFLP of the ITS1 gene amplicon which also clearly discriminated the patterns for *L donovani* and *L. major* as by Kumar et al.^12^ (Fig. 4B2). The pattern showed similarity with that of the reference strain LEM 138 *L. donovani* parasite. Species identification is a vital part of the diagnostic procedure, especially in areas where more than one species of *Leishmania* circulate^23^. Clinical manifestation of cutaneous leishmaniasis is determined not only by the infecting species but also by the individual immune response^24^. Therefore, species determination simply by observing the clinical presentation may not be enough. The presence of multiple species of *Leishmania* in a region demands the molecular identification to help for the determination of the clinical prognosis and select the most appropriate regimen to be administered to each individual^25^.

The amplified ITS1 PCR products were processed to the sequencing on seven samples (Four of *L. donovani* and three of *L. major*). The *L. donovani* sequences which were not grouped into a significant monophyletic cluster (only 62% bootstrap value) had significantly monophyletic (99% bootstrap value) among the *L. donovani* and *L. infantum* cluster. This distinction is the indication that the ITS1 marker is probably not sufficient to separate these extremely closely related species. Lower bootstrap value on the phylogenetic analysis with indication of paraphyletic cluster and 99.47% homology with *L. donovani* from the non-endemic state in southern India (GenBank Accession Number: MW053328.1) sequences is suggestive that the causative *L. donovani* species might be a new variant. So, further molecular analysis of these clinical isolates is needed to determine genetic makeup of the emerging atypical *L. donovani* variants. Our findings on *L. donovani* as the causative agent of cutaneous leishmaniasis is similar to other countries in Indian subcontinent like in Srilanka^26^ and Bhutan^27^ and India^28^. It would be an interesting study whether the *L. donovani* causing cutaneous leishmaniasis are of monophylectic cluster to the reported Indian subcontinent one. The information would be an interesting study for the other parts of the world as well where cutaneous leishmaniasis is gradually spreading to non-foci tropical and subtropical zones from temperate zone as observed in India^29^.

In this study, unexpectedly more cases of cutaneous leishmaniasis were detected in non-foci regions. Infections were seen in 16 districts encompassing all the seven provinces of Nepal spreading to all geographical Terai, Hill and Mountain regions. Interestingly, the higher cases were from hill (59%, 7 cases due to *L. donovani* and 6 by *L. major*) and mountain (32%; 6 cases due to *L. donovani* and 1 by *L. major*) regions situated at the latitude ranging from 27° 18′ 35.748′′ to 29° 59′ 12.192′′. The regions are in fact unusual foci for leishmaniasis and not yet identified as endemic regions by Kala Azar Elimination Program of Nepal Government for visceral leishmaniasis. Since country has not noted cutaneous leishmaniasis burden it has yet to realize those regions as potential endemic spots. Surprisingly, the least cases were detected from the overwhelmingly presumed Terai region (9%, 2 cases due to *L.major*). The infection in this region is further concerned issue as the causative species are only *L. major* despite the Terai region is endemic to visceral leishmaniasis caused by *L donovani*. The study results with confirmed cases due to *L major* (n = 9, 40.9%) and *L donovani* (n = 13, 59.1%) clearly showed that *L donovani* is the main etiological agent of cutaneous leishmaniasis in Nepal. Together the atypical cutaneous infections by *L donovani* parasite together with typical cutaneous infections by *L major* could be the reason for the increase of cutaneous leishmaniasis cases in Nepal. A larger and more in-depth study on cutaneous leishmaniasis may provide a broader picture of infection throughout Nepal.

The increase in the number of cases of cutaneous leishmaniasis and the spread of the disease to the high altitudes of the country could be due to multiple factors like genetic drift in parasites, shift of biological cell tropism, human mobility, climate change and vector's susceptibility for hosting multiple species. This study provides some answers. Among the 13 positive cases of cutaneous leishmaniasis caused by *L. donovani*, only one (CL27) had travel history to endemic country outside Nepal (United Arab Emirates) and none of the other positive cases caused by *L. donovani* had travelled to visceral leishmaniasis endemic places either inside or outside Nepal. This non-travel history strongly supports the higher chances of genetic drift within the circulating *L donovani* in Nepal making it more adaptive for skin tissue tropism. The ITS1 sequencing data and the phylogenetic analysis (paraphyletic cluster with* L donovani* having only 62% bootstrap value) has also backed up illustratively indicating the possibility of a new *L. donovani* variant which might have been emerged with a new manifestation of cutaneous disease in Nepal. However, the whole genome sequencing of the circulating parasites could provide more answers to contribute to a better understanding of this *L. donovani* evolution, occurrence of *Leishmania* species hybrids (as *L. major*/*L. donovani* or *L. donovani*/*L. donovani*) and/or adaptation to its new tissue tropism^30–34^.

It also seems that *L donovani* is an opportunistic parasite which uses the tissue tropism selecting the skin cells for better adaptation to cause cutaneous leishmaniasis. The three infected mountain districts which were tagged as doubtful districts of visceral leishmaniasis by the Nepal Government validate the opportunistic nature of the parasites. The infection response also suggests that *L donovani* has been indigenously circulating even in the cold mountain region with better adoption^35^. At the verge of visceral leishmaniasis elimination as public health problem in Nepal in the 18 endemic districts^36^, the opportunistic tissue tropism of *Leishmania* species remains an unresolved question. It implicates numerous factors requiring an understanding the genetic determinants and the biology of tropism with the consideration of molecular and cellular factors of host-parasite-vector interactions^34,37,38^. These cutaneous leishmaniasis cases due to *L. donovani* are attention-grabbing and should be considered as potential reservoirs that could unknowingly enhance the continuation of parasite transmission and a risk of resurgence of visceral leishmaniasis^36^. This study found that there is no human reservoir since the family and the neighbor of the infected patient have neither visceral leishmaniasis nor cutaneous leishmaniasis and post-kala-azar dermal leishmaniasis. There might be the animal reservoir responsible for the transmission of the parasite. In Nepal, domestic animals such as cows, buffalo, and goats as well as the stray dog can be reservoir host for *Leishmania spp*.^39,40^. Eight districts which were not targeted for Kala azar elimination have also been found infected with clear indication that there is need of urgent surveillance integrating in the campaigns of the surveillance of visceral leishmaniasis.

In recent past, the road facilities in Nepal have been notably increased making the travel easy and swift from endemic areas. The climate change due to global warming has shown direct effect in the rise in atmospheric temperature favoring the survival of the thermophilic vector in the higher altitudes^15,41,42^ and adopting the vectors for hosting multiple parasite species^43^. While analyzing on the potential transmission dimension due to travelling in endemic in-country and outside-country, it was detected that two cases (CL36 and CL39) had the travel history to the leishmaniasis endemic country (Saudi Arabia) where the subject had infection before returning to Nepal. Another case (CL-13) had travel history to hilly district, Darchula which is again categorized as doubtful endemic region. Both the cases were caused by *L. major* which is new species for Terai region where *L donovani* use to be pre-existing species causing visceral leishmaniasis. Hence, the study concludes that the *L. major* transmission in the Terai is via travel route of endemic regions. It indicates that Terai is yet not endemic to the cutaneous leishmaniasis but now onwards there is huge threat of quick indigenous circulation due to its favorable climate.

Genderwise disribution showed 59.09% (n = 13) male while 40.9% (n = 9) were female, most of the males were from the age group (21–40 yrs) and (41–60 yrs) which covers working age. In Nepal, females are mostly involved in household works and males are involved in outdoor works. This may have predisposed the males to the bite of sand fly. However, The gender-wise demography has not shown gender wise disparity (*P* = 0.666). The distribution of the disease in different age group was statistically not significant (*P* = 0.833), however age-wise prevalence showed that children-teenagers (< 20 yrs) and adult population (21–40 yrs) were most victimized. Since, the disease has been well known to cause skin lesions, mainly ulcers, leaving life-long scars and serious disability or psychosocial burden^44^. The present study showed morphology of the lesions showed that the most of the cases were localized (77.5%) and other were dispersed (22.5%), with 47.5% of the subjects affected in the facial part of the body followed by other exposed parts such as arms and neck. Therefore, the health issue should be addressed timely by diagnosing the cases promptly and appropriately before the localized lesions become dispersed and cases become severe.

Recently, World Health Organization (WHO) has published a new strategic framework for skin-related neglected tropical diseases to prevent, control, eliminate and eradicate of these diseases globally by 2030 including cutaneous leishmaniasis^3^. In the context of cutaneous leishmaniasis in Asia, the disease is present but uncommon^45^ as in Nepal. The progression of cutaneous leishmaniasis cases and geographic expansion of areas where both *L. major* and *L. donovani* is circulating must be taken into consideration. There would be many folds of population who might have been infected but could not reach to health centers for checkup due to different reasons. So, the percentile infection of this study may not represent the actual infection dimension in the country. It needs an active surveillance implemented which helps for actual epidemiological map. The Infectious and Viral Disease Research Laboratory (IVDRL) of Tribhuvan University of Nepal, which successfully adopted the molecular PCR-based test for diagnosis of cutaneous leishmaniasis in Nepal for the first time, could be exploited for diagnosis as well as for surveillance of cutaneous leishmaniasis all over the country using all the PCR laboratory set-ups during the Covid-19 pandemics. Country may develop centers for both the visceral and cutaneous leishmaniasis surveillance and control program in every district or province since, *L. donovani* is endemic in 18 districts and 50 districts listed in doubtful endemic ones^11^, and cutaneous leishmaniasis is seen in 16 districts of all provinces.

Further, cutaneous leishmaniasis disease and vector control programs should be launched at the earliest along with visceral leishmaniasis elimination program in order to implement leishmaniasis control management strategies as a whole. Accurate diagnostic tests should be implemented to detect and identify the causative *Leishmania* species of the disease, to help to avoid the misdiagnosis, to help clinicians to adapt suitable treatment, and update the distribution map of *Leishmania* species. To meet these needs, molecular diagnostic tools must be used for the diagnosis, understanding the emergence of new hotspots, control of reservoirs and *Leishmania* transmission. Hence, we strongly recommend the concerned health authorities to keep this validated kDNA minicircle PCR-based test in regular routine diagnosis of leishmaniasis for controlling the new emerging cutaneous disease in Nepal.

In conclusion, Nepal has a new threat of emerging cutaneous leishmaniasis with the concomitant presence of atypical skin infections due to *L. donovani* and typical due to *L. major*. The detected *L. donovani* species which do not show the monophyletic cluster with previously sequenced data (those having highest percentage identity and sequence similarity) indicated the need of mitochondrial and nuclear gene or whole genome sequencing to identify the possible different lineage of *L. donovani* circulating in Nepal. The transmission dynamics to the non-foci mountain and hill region is a serious concern not only for the Nepal but also for the whole World. Therefore, cutaneous leishmaniasis control program with kDNA based PCR diagnostic approach has to be commenced at the earliest.

## Methods

### Study area, patients and samples

The present study was conducted in Nepal between 2018 and 2020. Patients with cutaneous lesions presented to the three government hospitals, namely Sukraraj Tropical and Infectious Disease Hospital (STIDH)- Kathmandu, Bir Hospital- Kathmandu and Nepalganj Medical College and Teaching Hospital- Nepalganj, were taken care of for a clinical diagnosis and biopsies of the skin lesions were taken for routine diagnostic analyses. In this passive case-finding, the skin lesions were cleaned with 70% alcohol and 100 µL of normal saline injected to the lesion area or in some cases, slit skin was done using a sterile scalpel followed by tissue aspiration from periphery of each nodule/papule. For this study, 40 patients presented with cutaneous lesions suspected for cutaneous leishmaniasis were included. The tissue samples were used for microscopic examination, culture and molecular characterization.

### Microscopic examination and parasite culture

A small drop of undiluted tissue aspiration sample was used to prepare thin smear slides on the clean and grease free glass slides. The smear was left to air dry at room temperature. Fixation was done using 100% methanol for 30 s and rinsed with water followed by staining with 10% Giemsa for 30 min, rinsed off with tap water and allowed to get dry at room temperature. The slides were examined for amastigotes [Leishman–Donovan (LD) bodies] under 100X oil immersion in light microscope.

For culture, one to two drops lesion aspirates from each patient were inoculated to NNN tube aseptically. The cultured tubes were kept in BOD/cooling incubator at constant 26 °C for 9 days. The tubes were observed for growth of parasites from day-6 onwards in inverted microscope. After incubation period, the liquid media (~ 200 µL) of the culture having growth of parasites was inoculated to 25 cm^2^ culture flask containing 5 mL M199 complete media with 10% FBS and 0.5% streptomycin and penicillin (Gibco).

### DNA extraction and PCR amplification

DNA from each clinical tissue sample was extracted using Quick-DNA™ Miniprep Plus Kit (Zymo research), following the manufacturer’s instructions. By using the same method, DNA were also extracted from cultured parasites. The extracted DNA were kept at -20 °C until PCR analysis.

Two PCR assays were performed. For the first PCR assay, a kinetoplast minicircle DNA (kDNA) nested PCR was performed using CSB2F (C/GA/GTA/GCAGAAAC/TCCCGTTCA) and CSB1R (ATTTTTCG/CGA/TTTT/CGCAGAACG) primers for first round PCR (PCR-1), and 13Z (TCGCAGAACGCCCCT) and LiR (ACTGGGGGTTGGTGTAAAATAG) primers for second round PCR (PCR-2) as described by Noyes et al.^20^. Briefly, the PCR-1 consists of 94 °C for 2 min, followed by 40 cycles of amplification (94 °C for 50 s, 54 °C for 60 s, 72 °C for 90 s), and final extension at 72 °C for 10 min. In the PCR-2, 1 µL diluted PCR-1 product was used as template and same PCR conditions of PCR-1 were used, except annealing temperature which was increased from 54 to 56 °C. PCR amplicons were analyzed on 1.5% agarose gels. The 100 bp DNA ladder (Solis Biodyne) was used as the DNA molecular marker.

From DNA samples with a positive minicircle kDNA PCR result (from the second round PCR assay), an internal transcribed spacer-1 (ITS1) PCR was performed using LITSR (CTGGATCATTTTCCGATG) and L5.8S (TGATACCACTTATCGCAC) primers to amplify the ribosomal ITS region as described by Kumar et al.^12^. The ITS1 PCR conditions consists of 94 °C for 3 min, followed by 35 cycles (94 °C for 40 s, 52 °C for 50 s, 72 °C for 60 s), and final extension 72 °C for 10 min. The ITS1 PCR amplicons were analyzed on 1.5% agarose gels. The 100 bp DNA ladder (Solis Biodyne) was used as the DNA molecular marker.

### Restricted fragmentation length polymorphism (RFLP)

ITS1 PCR products (8–15 µL) were digested with HaeIII enzyme, according to the manufacturer’s instructions and analyzed on 3% agarose gels and visualized by UV light after being stained with ethidium bromide (0.3 µg/mL). The 25 bp DNA ladder (New England Biolab) was used as the DNA molecular marker.

### Sequencing and phylogenetic analysis

From seven PCR-confirmed cutaneous leishmaniasis patients, the ITS1 PCR products were purified using ExoSAP-IT Express (Affymetrix, Inc., CA, USA) according to the manufacturer’s manual. Briefly, 5 µL of PCR product and 2 µL of Exosap was mixed and incubated at 37 °C for 10 min and then at 80 °C for 10 min. The purified product was subjected to sequencing reactions using BigDye™ Terminator v3.1 Cycle Sequencing Kit (Applied Biosystems, CA, USA) following the manufacturer instructions. The Big Dye XterminatorTM Kit (Applied Biosystems) was used to further purify these single-stranded sequences. Finally, the purified amplicons were sequenced in an automated 3500XL Genetic Analyzer (Applied Biosystems)^46^.

The quality of raw sequences was checked in sequencer v. 5.0 (GeneCodes Corporation, MI, USA) through base calling and sequences were generated by using both strands consensus. For the present study, the ITS1 DNA sequences of the *Leishmania spp* parasites isolated from the seven PCR-confirmed cutaneous leishmaniasis patients were uploaded to National Center for Biotechnology Information (NCBI) and obtained respective accession numbers. For the identification of *Leishmania spp.*, the final sequence obtained from each sample was subjected to Nucleotide Basic Local Alignment Search Tool (BLAST) to search similarity with the *Leishmania spp.* sequences stored on NCBI database (Shrestha et al.^46^). Sequence alignment was performed by using the algorithm ClustalW; a Maximum Likelihood tree (ML tree) was constructed using the best model of multiple substitutions (i.e. the one presenting the Bayesian Information Criterion score (BIC score), and a statistical phylogeny test based on a bootstrap of 50,000 replicates was applied. The construction of ML tree was made with the software IQ-TREE v.2.2.0^47^ while alignment and tree editing were achieve by the MEGA7^48^.

### Statistical analysis and ethical statement

The data were statistically analyzed using Graph Pad Prism version 9.3.0 and SPSS version 21. Ethical approval was taken from Nepal Health Research Council. Written Informed Consent was taken from each patient informing the aim of the research, type of the study and method of sample collection before the enrollment in this research. In the case of minors, consents were given by their parents or guardians.

### Ethical approval

The ethical approval for the research involving human participants was granted from Nepal Health Research Council (NHRC) (Reg. No. 45/2018 and 114/2019). All the methods were carried out in accordance with NHRC guidelines and regulations. Informed consent was obtained from all participants and/or their legal guardians for publication of their anonymized demographic and clinical information, and laboratory findings.

## Supplementary Information

Supplementary Information.

## Data Availability

The gene sequences of the sequence of ITS1 region obtained in this study were deposited with GenBank Accession Number: MT023531.1 and GenBank Accession Number: MW053328.1 corresponding *L. major* and *L. donovani* respectively. Multiple sequence alignment of ITS1 and nucleotide substitution carried by the best Bayesian Information Criterion score of representative parasite isolates from patients with those of *L. donovani* complex and *L. major* reference strains (Supplementary data S-IV). Visceral leishmaniasis (VL) endemic district of Nepal described in National Guideline on Kala-azar Elimination Program 2019 is available in the Nepal Government’s EDCD (Epidemiology and Disease Control Division) web page http://edcd.gov.np/uploads/resource/5caca993d18db.pdf. Similarly the Endemicity status of foreign country can be viewed in WHO’s webpage https://apps.who.int/neglected_diseases/ntddata/leishmaniasis/leishmaniasis.html.
