# Supplementary material for: Leishmania donovani persistence and circulation causing cutaneous leishmaniasis in unusual-foci of Nepal
Source: Sci Rep. 2023 Jul 29;13:12329. doi: 10.1038/s41598-023-37458-6 (PMC10387047; doi:10.1038/s41598-023-37458-6)
Supplement: Supplementary file 1 — Supplementary Information. [file 41598_2023_37458_MOESM1_ESM.pdf]

Supplementary Figures and Tables

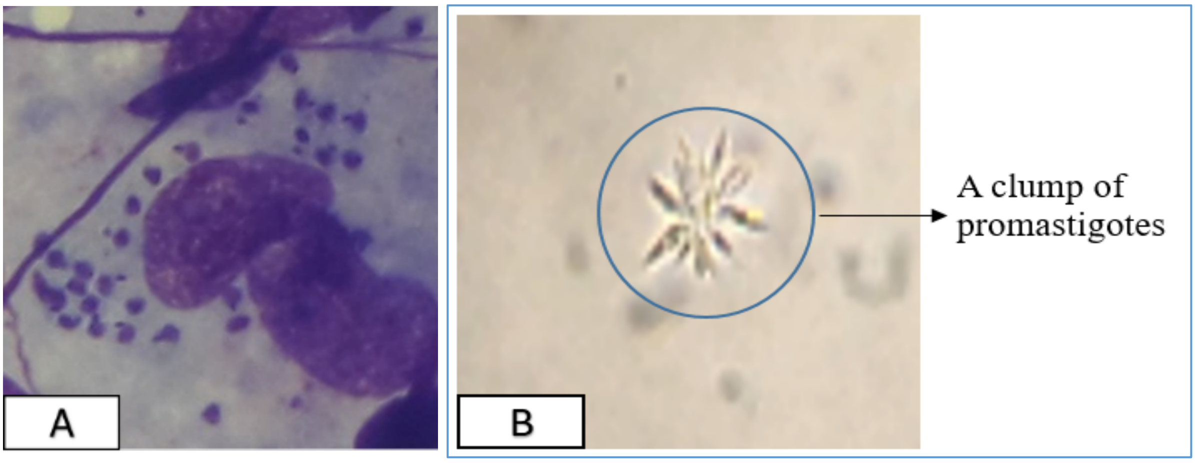

Supplementary Figure S-I: Whole parasite visualization as microscopic and culture diagnosis. Giemsa stained parasite (A) and culture of parasite in NNN media (B)

Supplementary Table S-II: Clinical manifestations of the cutaneous lesions

Geographic Region	Province no. (Name)	District	VL Endemicity	GPS coordinates of district's center**		CL positive cases ***	General information of patient		CL etiology		National VL elimination program lunched district	Travel history of patient outside Nepal		Travel history of patient inside Nepal		Medication used before sampling
				Latitude	Longitude		Age (year)	Gender	*L. major*	*L. donovani*		Country	Status of endemicity	Inside country	Nepal's Kalaazar endemic district****	
Hill	1 (Koshi)	Bhojpur	Endemic	27°21'32.868"	87°8'5.136"	1	43	M		CL27	Yes	UAE Dubai	No cases	No		Antifungal
		Okhaldgunga	Endemic	27°18'35.748"	86°24'31.608"	1	58	M	CL29		Yes	No		No		No medication
	3(Bagmati)	Ramechhap	Doubtful endemic	27°29'11.868"	85°51'6.912"	1	15	M	CL12		No	No		No		Antibiotic and Antifungal
	4(Gandaki)	Tanahun	Doubtful endemic	27°53'18.348"	83°58'28.992"	1	85	M	CL8		No	No		No		Antibiotic
		Syangja	Doubtful endemic	27°53'32.82"	83°48'45.612"	1	67	F		CL10	No	UK	No	No		NA
		Gorkha*	Doubtful endemic	27°59'9.276"	84°37'34.392"	2	54	F	CL17		No	No		No		NA
							66	F	CL23		No	No		Yes, Chitwan	Doubtful endemic	NA
		Baglung	Non-endemic	28°24'36.648"	83°0'7.956"	1	48	M	CL36		No	Saudi Arabia	Yes(CL,VL)	No		NA
	5 (Lumbini)	Palpa	Endemic	27°43'13.548"	83°38'21.66"	1	23	M		CL15	Yes	India	Yes(CL,VL)	No		Antibiotic, Antifungal and Antileishmanial
		Rukum*	Doubtful endemic	28°45'39.312"	82°27'39.456"	1	20	M		CL35	No	No		Yes, Dang	Doubtful endemic	NA
	6 (Karnali)	Jajarkot*	Doubtful endemic	28°43'51.6"	82°12'10.26"	1	35	M		CL22	No	No		No		Antifungal
	7 (Sudur Pashchim)	Baitadi	Doubtful endemic	29°33'54.072"	80°23'55.752"	2	54	F		CL14	No	No		No		Antituberculosis
							60	F		CL25	No	No		No		Antifungal
Mountain	6. (Karnali)	Humla	Doubtful endemic	29°59'12.192"	81°49'59.34"	1	33	M		CL16	No	No		No		Antibiotic and Antifungal
		Kalikot*	Doubtful endemic	29°12'49.896"	81°35'23.532"	3	19	F		CL2	No	No		Yes, Pyuthan	Doubtful endemic	NA
							9	F	CL5		No	No		Yes, Surkhet	Yes	Antifungal
							61	M		CL32	No	No		No		NA
	7 (Sudur	Bajura	Doubtful endemic	29°31'47.568"	81°46'59.268"	3	16	F		CL18	No	No		No		NA
							15	F		CL19	No	No		No		NA
							11	M		CL40	No	No		No		No medication
Terai	1	Jhapa	Endemic	26°36'25.308"	87°44'41.748"	1	55	M	CL39		Yes	Saudi Arabia	Yes(CL,VL)	No		NA
	2 (Madhesh Pradesh)	Rautahut	Endemic	26°58'9.156"	85°15'37.296"	1	26	M	CL13		Yes	No		Yes, Darchula	Doubtful endemic	Antifungal

*District spread with hilly and mountainous areas. **GPS coordinates the center of the districts. ***Number of cases with positive parasitological and molecular diagnosis or only molecular diagnosis. *****Visceral leishmaniasis (VL) endemic district of Nepal described in National Guideline on Kala-azar Elimination Program 2019 (http://www.edcd.gov.np/resource-detail/national-guideline-on-kala-azar-elimination-program-2019); Endemicity status of foreign country defined by WHO according to the reported*

cases (*https://apps.who.int/neglected_diseases/ntddata/leishmaniasis/ leishmaniasis.html*). Male (M); Female (F); NA: Not Available; Shaded cells: No travel history.

Supplementary Table S-III: Clinical manifestations of the cutaneous lesions

Site of Lesion	Frequency	Number of lesion			Type of lesion		Dispersion of the lesion	
		1	2	>3	Dry	Moist	Localized	Dispersed
Face	19	13	3	3	16	3	16	3
Neck	5	5			2	3	4	1
Hand	5	3	1	1	4	1	3	2
Leg	3	2		1		3	3	
Abdominal part or body	3	1		2	3			3
Face and Hand	2		1	1		2	2	
Hand and Leg	1			1		1	1	
Hand and abdomen	1		1		1		1	
Face, Hand and Leg	1			1		1	1	
Total	40	24	6	10	26	14	31	9

Supplementary data S-IV: Multiple sequence alignment of ITS1 of representative parasite isolates from patients with those of *L. donovani* complex and *L. major* reference strains.

				JQ730001.1 L. donovani isolate donov	MW053322.1 L. donov	OM220625.1 L. donovani isolate MW23 donov	OM220617.1 L. donovani isolate donov	MK211306.1 L. donovani isolate TM- donov	MW053324.1 L. donov	MW053328.1 L. donov	CL18 (ON146457) donov	CL22 (ON146458) donov	CL25 (ON146459) donov	CL40 (ON146460) donov		KP691595.1 L. tropica tropica	OK338429.1 L. tropica isolate 356/TN/19 tropica	OK354361.1 L. tropica isolate 393/TN/19 tropica	KY963130.1 L. tropica tropica	KY963132.1 L. tropica tropica	KX096623.1 L. infantum isolate 2013/MO13 infantu	MN422062.1 L. infantum infantu	MN503524.1 L. infantum infantu	MN503525.1 L. infantum infantu	MN503526.1 L. infantum infantu	MN503527.1 L. infantum infantu	AY573187.1 L. major strain major	MT023531.1 L. major voucher MK-MZ-DM4 major	KX821679.1 L. major strain major	CL 17 (ON146453) major	CL23 (ON146454) major	CL29tissue major	CL29culture major
1			1	A	A	A	A	A	A	A	A	A	A	A		A	A	A	A	A	A	A	A	A	A	A	A	A	A	A	A	A	A
2			2	T	T	T	T	T	T	T	T	T	T	T		T	T	T	T	T	T	T	T	T	T	T	T	T	T	T	T	T	T
3			3	T	T	T	T	T	T	T	T	T	T	T		T	T	T	T	T	T	T	T	T	T	T	T	T	T	T	T	T	T
4			4	A	A	A	A	A	A	A	A	A	A	A		A	A	A	A	A	A	A	A	A	A	A	A	A	A	A	A	A	A
5			5	C	C	C	C	C	C	C	C	C	C	C		C	C	C	C	C	C	C	C	C	C	C	C	C	C	C	C	C	C
6			6	A	A	A	A	A	A	A	A	A	A	A		A	A	A	A	A	A	A	A	A	A	A	A	A	A	A	A	A	A
7			7	C	C	C	C	C	C	C	C	C	C	C		C	C	C	C	C	C	C	C	C	C	C	C	C	C	C	C	C	C
8			8	C	C	C	C	C	C	C	C	C	C	C		C	C	C	C	C	C	C	C	C	C	C	C	C	C	C	C	C	C
9			9	A	A	A	A	A	A	A	A	A	A	A		C	C	C	C	C	C	C	C	C	C	C	C	C	C	C	C	C	C
10		1	0	A	A	A	A	A	A	A	A	A	A	A		-	-	-	-	-	-	-	-	-	-	-	C	C	C	C	C	C	C
11		1	1	A	A	A	A	A	A	A	A	A	A	A		A	A	A	A	A	-	-	-	-	-	-	A	A	A	A	A	A	A
12		1	2	A	A	A	A	A	A	A	A	A	A	A		A	A	A	A	A	A	A	A	A	A	A	A	A	A	A	A	A	A
13		1	3	A	A	A	A	A	A	A	A	A	A	A		A	A	A	A	A	A	A	A	A	A	A	A	A	A	A	A	A	A
14		1	4	A	A	A	A	A	A	A	A	A	A	A		A	A	A	A	A	A	A	A	A	A	A	A	A	A	A	A	A	A
15		1	5	A	A	A	A	A	A	A	A	A	A	A		A	A	A	A	A	A	A	A	A	A	A	A	A	A	A	A	A	A
16		1	6	A	A	A	A	A	A	A	A	A	A	A		A	A	A	A	A	A	A	A	A	A	A	A	A	A	A	A	A	A
17		1	7	-	-	-	-	-	-	-	A	A	A	A		A	A	A	A	A	A	A	A	A	A	A	-	-	-	A	A	A	A
18		1	8	C	C	C	C	C	C	C	C	C	C	C		C	C	C	C	C	C	C	C	C	C	C	C	C	C	C	C	C	C
19		1	9	A	A	A	A	A	A	A	A	A	A	A		A	A	A	A	A	A	A	A	A	A	A	A	A	A	A	A	A	A
20		2	0	T	T	T	T	T	T	T	T	T	T	T		T	T	T	T	T	T	T	T	T	T	T	T	T	T	T	T	T	T
21		2	1	A	A	A	A	A	A	A	A	A	A	A		A	A	A	A	A	A	A	A	A	A	A	A	A	A	A	A	A	A
22		2	2	T	T	T	T	T	T	T	T	T	T	T		T	T	T	T	T	T	T	T	T	T	T	T	T	T	T	T	T	T
23		2	3	A	A	A	A	A	A	A	A	A	A	A		A	A	A	A	A	A	A	A	A	A	A	A	A	A	A	A	A	A
24		2	4	C	C	C	C	C	C	C	C	C	C	C		C	C	C	C	C	C	C	C	C	C	C	C	C	C	C	C	C	C
25		2	5	-	-	-	-	-	-	-	-	-	-	-		A	A	A	A	-	-	-	-	-	-	-	-	-	-	-	-	-	-
26		2	6	-	-	-	-	-	-	-	-	-	-	-		A	A	A	A	-	-	-	-	-	-	-	-	-	-	-	-	-	-
27		2	7	A	A	A	A	A	A	A	A	A	A	A		A	A	A	A	A	A	A	A	A	A	A	A	A	A	A	A	A	A
28		2	8	A	A	A	A	A	A	A	A	A	A	A		A	A	A	A	A	A	A	A	A	A	A	A	A	A	A	A	A	A
29		2	9	C	C	C	C	C	C	C	C	C	C	C		C	C	C	C	C	C	C	C	C	C	C	C	C	C	C	C	C	C
30		3	0	T	T	T	T	T	T	T	T	T	T	T		T	T	T	T	T	T	T	T	T	T	T	T	T	T	T	T	T	T
31		3	1	C	C	C	C	C	C	C	C	C	C	C		C	C	C	C	C	C	C	C	C	C	C	C	C	C	C	C	C	C
32		3	2	G	G	G	G	G	G	G	G	G	G	G		G	G	G	G	G	G	G	G	G	G	G	G	G	G	G	G	G	G
33		3	3	G	G	G	G	G	G	G	G	G	G	G		G	G	G	G	G	G	G	G	G	G	G	G	G	G	G	G	G	G
34		3	4	G	G	G	G	G	G	G	G	G	G	G		G	G	G	G	G	G	G	G	G	G	G	G	G	G	G	G	G	G
35		3	5	G	G	G	G	G	G	G	G	G	G	G		G	G	G	G	G	G	G	G	G	G	G	G	G	G	G	G	G	G
36		3	6	A	A	A	A	A	A	A	A	A	A	A		A	A	A	A	A	A	A	A	A	A	A	A	A	A	A	A	A	A
37		3	7	G	G	G	G	G	G	G	G	G	G	G		G	G	G	G	G	G	G	G	G	G	G	G	G	G	G	G	G	G
38		3	8	A	A	A	A	A	A	A	A	A	A	A		G	G	G	G	G	A	A	A	A	A	A	G	G	G	G	G	G	G
39		3	9	C	C	C	C	C	C	C	C	C	C	C		C	C	C	C	C	C	C	C	C	C	C	C	C	C	C	C	C	C
40		4	0	C	C	C	C	C	C	C	C	C	C	C		C	C	C	C	C	C	C	C	C	C	C	T	T	T	T	T	T	T
41		4	1	T	T	T	T	T	T	T	T	T	T	T		T	T	Y	T	T	T	T	T	T	T	T	T	T	T	T	T	T	T
42		4	2	A	A	A	A	A	A	A	A	A	A	A		A	A	A	A	A	A	A	A	A	A	A	A	A	A	A	A	A	A
43		4	3	T	T	T	T	T	T	T	T	T	T	T		T	T	T	T	T	T	T	T	T	T	T	T	T	T	T	T	T	T
44		4	4	G	G	G	G	G	G	G	G	G	G	G		A	A	A	A	A	G	G	G	G	G	G	T	T	T	T	T	T	T
45		4	5	-	-	-	-	-	-	-	-	-	-	-		-	-	-	-	-	-	-	-	-	-	-	C	C	C	C	C	C	C
46		4	6	T	T	T	T	T	T	T	T	T	T	T		T	T	T	T	T	T	T	T	T	T	T	T	T	T	T	T	T	T
47		4	7	A	A	A	A	A	A	A	A	A	A	A		A	A	A	A	A	A	A	A	A	A	A	A	A	A	A	A	A	A
48		4	8	T	T	T	T	T	T	T	T	T	T	T		T	T	T	T	T	T	T	T	T	T	T	T	T	T	T	T	T	T
49		4	9	A	A	A	A	A	A	A	A	A	A	A		A	A	A	A	A	A	A	A	A	A	A	A	A	A	A	A	A	A
50		5	0	T	T	T	T	T	T	T	T	T	T	T		T	T	T	T	T	T	T	T	T	T	T	T	T	T	T	T	T	T
51		5	1	A	A	A	A	A	A	A	A	A	A	A		T	T	T	T	T	A	A	A	A	A	A	A	A	A	A	A	A	A
52		5	2	A	A	-	-	-	A	A	A	A	A	A		A	A	A	A	A	-	-	-	-	-	-	-	-	-	-	-	-	-
53		5	3	T	T	T	T	T	T	T	T	T	T	T		T	T	T	T	T	-	-	-	-	-	-	T	T	T	T	T	T	T
54		5	4	A	A	A	A	A	A	A	A	A	A	A		A	A	A	A	A	-	-	-	-	-	-	A	A	A	A	A	A	A
55		5	5	T	T	T	T	-	T	T	T	T	T	T		C	C	C	C	C	-	-	-	-	-	-	T	T	T	T	T	T	T
56		5	6	A	A	A	A	-	A	A	A	A	A	A		A	A	A	A	A	-	-	-	-	-	-	A	A	A	A	A	A	A
57		5	7	-	-	-	-	-	-	-	-	-	-	-		T	T	T	T	T	-	-	-	-	-	-	T	T	T	-	-	-	-
58		5	8	T	T	T	T	T	T	T	T	T	T	T		T	T	T	T	T	T	T	T	T	T	T	A	A	A	-	-	-	-
59		5	9	A	A	A	A	A	A	A	A	A	A	A		A	A	A	A	A	A	A	A	A	A	A	G	G	G	G	G	G	G
60		6	0	T	T	T	T	T	T	T	T	T	T	T		T	T	T	T	T	T	T	T	T	T	T	T	T	T	T	T	T	T

				JQ730001.1 L. donovani isolate Trashigang1	MW053322.1 L. donovani	OM220625.1 L. donovani isolate MW23	OM220617.1 L. donovani isolate PK604	MK211306.1 L. donovani isolate TM-222 BD 2014	MW053324.1 L. donovani	MW053328.1 L. donovani	CL18 (ON146457)	CL22 (ON146458)	CL25 (ON146459)	CL40 (ON146460)		KP691595.1 L. tropica isolate	OK338429.1 L. tropica isolate 356/TN/19	OK354361.1 L. tropica isolate 393/TN/19	KY963130.1 L. tropica isolate	KY963132.1 L. tropica isolate	KX096623.1 L. infantum isolate 2013/MO13	MN422062.1 L. infantum	MN503524.1 L. infantum	MN503525.1 L. infantum	MN503526.1 L. infantum	MN503527.1 L. infantum	AY573187.1 L. major strain MHOM/IR/00/PII2	MT023531.1 L. major voucher MK-MZ-DM4	KX821679.1 L. major strain MHOM/AT/16/AS16	CL17 (ON146453)	CL23 (ON146454)	CL29tissue (ON146455)	CL29culture (ON146456)
				dono	dono	dono	dono	dono	dono	dono	dono	dono	dono	dono		tropica	tropica	tropica	tropica	tropica	infantu	infantu	infantu	infantu	infantu	infantu	major	major	major	major	major	major	major
61		6	1	G	G	G	G	G	G	G	G	G	G	G		A	A	A	A	A	G	G	G	G	G	G	A	A	A	A	A	A	A
62		6	2	T	T	T	T	T	T	T	T	T	T	T		T	T	T	T	T	T	T	T	T	T	T	T	T	T	T	T	T	T
63		6	3	A	A	A	A	A	A	A	A	A	A	A		A	A	A	A	A	A	A	A	A	A	A	A	A	A	A	A	A	A
64		6	4	G	G	G	G	G	G	G	G	G	G	G		G	G	G	G	G	G	G	G	G	G	G	G	G	G	G	G	G	G
65		6	5	G	G	G	G	G	G	G	G	G	G	G		G	G	G	G	G	G	G	G	G	G	G	G	G	G	G	G	G	G
66		6	6	C	C	C	C	C	C	C	C	C	C	C		C	C	C	C	C	C	C	C	C	C	C	C	C	C	C	C	C	C
67		6	7	C	C	C	C	C	C	C	C	C	C	C		C	C	C	C	C	C	C	C	C	C	C	T	T	T	T	T	T	T
68		6	8	T	T	T	T	T	T	T	T	T	T	T		T	T	T	T	T	T	T	T	T	T	T	T	T	T	T	T	T	T
69		6	9	T	T	T	T	T	T	T	T	T	T	T		T	T	T	T	T	T	T	T	T	T	T	T	T	T	T	T	T	T
70		7	0	T	T	T	T	T	T	T	T	T	T	T		T	T	T	T	T	T	T	T	T	T	T	T	T	T	T	T	T	T
71		7	1	C	C	C	C	C	C	C	C	C	C	C		C	C	C	C	C	C	C	C	C	C	C	C	C	C	C	C	C	C
72		7	2	C	C	C	C	C	C	C	C	C	C	C		C	C	C	C	C	C	C	C	C	C	C	C	C	C	C	C	C	C
73		7	3	-	-	-	-	-	-	-	-	-	-	-		C	C	C	C	C	-	-	-	-	-	-	-	-	-	-	-	-	-
74		7	4	-	-	-	-	-	-	-	-	-	-	-		A	A	A	A	A	-	-	-	-	-	-	-	-	-	-	-	-	-
75		7	5	C	C	C	C	C	C	C	C	C	C	C		C	C	C	C	C	C	C	C	C	C	C	C	C	C	C	C	C	C
76		7	6	A	A	A	A	A	A	A	A	A	A	A		A	A	A	A	A	A	A	A	A	A	A	A	A	A	A	A	A	A
77		7	7	C	C	C	C	C	C	C	C	C	C	C		C	C	C	C	C	C	C	C	C	C	C	C	C	C	C	C	C	C
78		7	8	A	A	A	A	A	A	A	A	A	A	A		A	A	A	A	A	A	A	A	A	A	A	A	A	A	A	A	A	A
79		7	9	T	T	T	T	T	T	T	T	T	T	T		T	T	T	T	T	T	T	T	T	T	T	T	T	T	T	T	T	T
80		8	0	A	A	A	A	A	A	A	A	A	A	A		A	A	A	A	A	A	A	A	A	A	A	A	A	A	A	A	A	A
81		8	1	C	C	C	C	C	C	C	C	C	C	C		C	C	C	C	C	C	C	C	C	C	C	C	C	C	C	C	C	C
82		8	2	A	A	A	A	A	A	A	A	A	A	A		A	A	A	A	A	A	A	A	A	A	A	A	A	A	A	A	A	A
83		8	3	C	C	C	C	C	C	C	C	C	C	C		C	C	C	C	C	C	C	C	C	C	C	C	C	C	C	C	C	C
84		8	4	A	A	A	A	A	A	A	A	A	A	A		A	A	A	A	A	A	A	A	A	A	A	A	A	A	A	A	A	A
85		8	5	G	G	G	G	G	G	G	G	G	G	G		G	G	G	G	G	G	G	G	G	G	G	G	G	G	G	G	G	G
86		8	6	C	C	C	C	C	C	C	C	C	C	C		C	C	C	C	C	C	C	C	C	C	C	C	C	C	C	C	C	C
87		8	7	A	A	A	A	A	A	A	A	A	A	A		A	A	A	A	A	A	A	A	A	A	A	A	A	A	A	A	A	A
88		8	8	A	A	A	A	A	A	A	A	A	A	A		A	A	A	A	A	A	A	A	A	A	A	A	A	A	A	A	A	A
89		8	9	A	A	A	A	A	A	A	A	A	A	A		A	A	A	A	A	A	A	A	A	A	A	A	A	A	A	A	A	A
90		9	0	G	G	G	G	G	G	G	G	G	G	G		C	C	C	C	C	G	G	G	G	G	G	C	C	C	C	C	C	C
91		9	1	T	T	T	T	T	T	T	T	T	T	T		T	T	T	T	T	T	T	T	T	T	T	T	T	T	T	T	T	T
92		9	2	T	T	T	T	T	T	T	T	T	T	T		T	T	T	T	T	T	T	T	T	T	T	T	T	T	T	T	T	T
93		9	3	T	T	T	T	T	T	T	T	T	T	T		T	T	T	T	T	T	T	T	T	T	T	T	T	T	T	T	T	T
94		9	4	T	T	T	T	T	T	T	T	T	T	T		T	T	T	T	T	T	T	T	T	T	T	T	T	T	T	T	T	T
95		9	5	G	G	G	G	G	G	G	G	G	G	G		G	G	G	G	G	G	G	G	G	G	G	A	A	A	A	A	A	A
96		9	6	T	T	T	T	T	T	T	T	T	T	T		T	T	T	T	T	T	T	T	T	T	T	T	T	T	T	T	T	T
97		9	7	A	A	A	A	A	A	A	A	A	A	A		A	A	A	A	A	A	A	A	A	A	A	A	A	A	A	A	A	A
98		9	8	C	C	C	C	C	C	C	C	C	C	C		C	C	C	C	C	C	C	C	C	C	C	C	C	C	C	C	C	C
99		9	9	T	T	T	T	T	T	T	T	T	T	T		T	T	T	T	T	T	T	T	T	T	T	T	T	T	T	T	T	T
100	1	0	0	C	C	C	C	C	C	C	C	C	C	C		C	C	C	C	C	C	C	C	C	C	C	C	C	C	C	C	C	C
101	1	0	1	A	A	A	A	A	A	A	A	A	A	A		G	G	G	G	G	A	A	A	A	A	A	A	A	A	A	A	A	A
102	1	0	2	A	A	A	A	A	A	A	A	A	A	A		A	A	A	A	A	A	A	A	A	A	A	A	A	A	A	A	A	A
103	1	0	3	A	A	A	A	A	A	A	A	A	A	A		A	A	A	A	A	A	A	A	A	A	A	A	A	A	A	A	A	A
104	1	0	4	A	A	A	A	A	A	A	A	A	A	A		G	G	G	G	G	A	A	A	A	A	A	A	A	A	A	A	A	A
105	1	0	5	T	T	T	T	T	T	T	T	T	T	T		T	T	T	T	T	T	T	T	T	T	T	T	T	T	T	T	T	T
106	1	0	6	T	T	T	T	T	T	T	T	T	T	T		T	T	T	T	T	T	T	T	T	T	T	T	T	T	T	T	T	T
107	1	0	7	T	T	T	T	T	T	T	T	T	T	T		T	T	T	T	T	T	T	T	T	T	T	T	T	T	T	T	T	T
108	1	0	8	G	G	G	G	G	G	G	G	G	G	G		G	G	G	G	G	G	G	G	G	G	G	G	G	G	G	G	G	G
109	1	0	9	C	C	C	C	C	C	C	C	C	C	C		C	C	C	C	C	C	C	C	C	C	C	C	C	C	C	C	C	C
110	1	1	0	A	A	A	A	A	A	A	A	A	A	A		A	A	A	A	A	A	A	A	A	A	A	A	A	A	A	A	A	A
111	1	1	1	G	G	G	G	G	G	G	G	G	G	G		G	G	G	G	G	G	G	G	G	G	G	G	G	G	G	G	G	G
112	1	1	2	T	T	T	T	T	T	T	T	T	T	T		T	T	T	T	T	T	T	T	T	T	T	T	T	T	T	T	T	T
113	1	1	3	A	A	A	A	A	A	A	A	A	A	A		A	A	A	A	A	A	A	A	A	A	A	A	A	A	A	A	A	A
114	1	1	4	A	A	A	A	A	A	A	A	A	A	A		A	A	A	A	A	A	A	A	A	A	A	A	A	A	A	A	A	A
115	1	1	5	A	A	A	A	A	A	A	A	A	A	A		A	A	A	A	A	A	A	A	A	A	A	-	-	-	-	-	-	-
116	1	1	6	A	A	A	A	-	A	A	A	A	A	A		C	C	C	C	C	A	A	A	A	A	A	-	-	-	-	-	-	-
117	1	1	7	A	A	A	A	A	A	A	A	A	A	A		A	A	A	A	A	A	A	A	A	A	A	A	A	A	A	A	A	A
118	1	1	8	A	A	A	A	A	A	A	A	A	A	A		A	A	A	A	A	A	A	A	A	A	A	A	A	A	A	A	A	A
119	1	1	9	A	A	A	A	A	A	A	A	A	A	A		A	A	A	A	A	A	A	A	A	A	A	A	A	A	A	A	A	A
120	1	2	0	A	A	A	A	A	A	A	A	A	A	A		A	A	A	A	A	A	A	A	A	A	A	A	A	A	A	A	A	A

				JQ730001.1 L. donovani isolate Trashigang1	MW053322.1 L. donovani isolate 3	OM220625.1 L. donovani isolate MW23	OM220617.1 L. donovani isolate PK604	MK211306.1 L. donovani isolate TM-222 BD 2014 SB	MW053324.1 L. donovani isolate 5	MW053328.1 L. donovani isolate 9	CL18 (ON146457)	CL22 (ON146458)	CL25 (ON146459)	CL40 (ON146460)		KP691595.1 L. tropica isolate GSKH 1	OK338429.1 L. tropica isolate 356/TN/19	OK354361.1 L. tropica isolate 393/TN/19	KY963130.1 L. tropica isolate Pv76	KY963132.1 L. tropica isolate PvMBF	KX096623.1 L. infantum isolate 2013/MO13	MN422062.1 L. infantum isolate 35	MN503524.1 L. infantum isolate 37	MN503525.1 L. infantum isolate 42	MN503526.1 L. infantum isolate 29	MN503527.1 L. infantum isolate 23	AY573187.1 L. major strain MHOM/IR/00/PII2	MT023531.1 L. major voucher MK-MZ-DM4	KX821679.1 L. major strain MHOM/AT/16/AS16	CL 17 (ON146453)	CL23 (ON146454)	CL29tissue (ON146455)	CL29culture (ON146456)
				donovani	donovani	donovani	donovani	donovani	donovani	donovani	donovani	donovani	donovani	donovani		tropica	tropica	tropica	tropica	tropica	infantum	infantum	infantum	infantum	infantum	infantum	major	major	major	major	major	major	major
121	1	2	1	G	G	G	G	G	G	G	G	G	G	G		G	G	G	G	G	G	G	G	G	G	G	G	G	G	G	G	G	G
122	1	2	2	G	G	G	G	G	G	G	G	G	G	G		G	G	G	G	G	G	G	G	G	G	G	G	G	G	G	G	G	G
123	1	2	3	C	C	C	C	C	C	C	C	C	C	C		C	C	C	C	C	C	C	C	C	C	C	C	C	C	C	C	C	C
124	1	2	4	C	C	C	C	C	C	C	C	C	C	C		C	C	C	C	C	C	C	C	C	C	C	C	C	C	C	C	C	C
125	1	2	5	G	G	G	G	G	G	G	G	G	G	G		G	G	G	G	G	G	G	G	G	G	G	G	G	G	G	G	G	G
126	1	2	6	A	A	A	A	A	A	A	A	A	A	A		A	A	A	A	A	A	A	A	A	A	A	A	A	A	A	A	A	A
127	1	2	7	T	T	T	T	T	T	T	T	T	T	T		T	T	T	T	T	T	T	T	T	T	T	T	T	T	T	T	T	T
128	1	2	8	C	C	C	C	C	C	C	C	C	C	C		C	C	C	C	C	C	C	C	C	C	C	C	C	C	C	C	C	C
129	1	2	9	G	G	G	G	G	G	G	G	G	G	G		G	G	G	G	G	G	G	G	G	G	G	G	G	G	G	G	G	G
130	1	3	0	A	A	A	A	A	A	A	A	A	A	A		A	A	A	A	A	A	A	A	A	A	A	A	A	A	A	A	A	A
131	1	3	1	C	C	C	C	C	C	C	C	C	C	C		C	C	C	C	C	C	C	C	C	C	C	C	C	C	C	C	C	C
132	1	3	2	G	G	G	G	G	G	G	G	G	G	G		G	G	G	G	G	G	G	G	G	G	G	G	G	G	G	G	G	G
133	1	3	3	T	T	T	T	T	T	T	T	T	T	T		T	T	T	T	T	T	T	T	T	T	T	T	T	T	T	T	T	T
134	1	3	4	T	T	T	T	T	T	T	T	T	T	T		T	T	T	T	T	T	T	T	T	T	T	T	T	T	T	T	T	T
135	1	3	5	A	A	A	A	A	A	A	A	A	A	A		A	A	A	A	A	A	A	A	A	A	A	G	G	G	G	G	G	G
136	1	3	6	T	T	T	T	T	T	T	T	T	T	T		T	T	T	T	T	T	T	T	T	T	T	T	T	T	T	T	T	T
137	1	3	7	-	-	-	-	-	-	-	-	-	-	-		-	-	-	-	-	-	-	-	-	-	-	A	A	A	A	A	A	A
138	1	3	8	-	-	-	-	-	-	-	-	-	-	-		-	-	-	-	-	-	-	-	-	-	-	G	G	G	G	G	G	G
139	1	3	9	A	A	A	A	A	A	A	A	A	A	A		A	A	A	A	A	A	A	A	A	A	A	A	A	A	A	A	A	A
140	1	4	0	A	A	A	A	A	A	A	A	A	A	A		A	A	A	A	A	A	A	A	A	A	A	A	A	A	A	A	A	A
141	1	4	1	C	C	C	C	C	C	C	C	C	C	C		C	C	C	C	C	C	C	C	C	C	C	C	C	C	C	C	C	C
142	1	4	2	G	G	G	G	G	G	G	G	G	G	G		G	G	G	G	G	G	G	G	G	G	G	G	G	G	G	G	G	G
143	1	4	3	C	C	C	C	C	C	C	C	C	C	C		C	C	C	C	C	C	C	C	C	C	C	C	C	C	C	C	C	C
144	1	4	4	A	A	A	A	A	A	A	A	A	A	A		A	A	A	A	A	A	A	A	A	A	A	A	A	A	A	A	A	A
145	1	4	5	C	C	C	C	C	C	C	C	C	C	C		C	C	C	C	C	C	C	C	C	C	C	C	C	C	C	C	C	C
146	1	4	6	C	C	C	C	C	C	C	C	C	C	C		C	C	C	C	C	C	C	C	C	C	C	C	C	C	C	C	C	C
147	1	4	7	G	G	G	G	G	G	G	G	G	G	G		G	G	G	G	G	G	G	G	G	G	G	G	G	G	G	G	G	G
148	1	4	8	C	C	C	C	C	C	C	C	C	C	C		C	C	C	C	C	C	C	C	C	C	C	C	C	C	C	C	C	C
149	1	4	9	C	C	C	C	C	C	C	C	C	C	C		C	C	C	C	C	C	C	C	C	C	C	C	C	C	C	C	C	C
150	1	5	0	T	T	T	T	T	T	T	T	T	T	T		T	T	T	T	T	T	T	T	T	T	T	T	T	T	T	T	T	T
151	1	5	1	A	A	A	A	A	A	A	A	A	A	A		A	A	A	A	A	A	A	A	A	A	A	A	A	A	A	A	A	A
152	1	5	2	T	T	T	T	T	T	T	T	T	T	T		T	T	T	T	T	T	T	T	T	T	T	T	T	T	T	T	T	T
153	1	5	3	-	-	-	-	-	-	-	-	-	-	-		A	A	A	A	A	-	-	-	-	-	-	A	A	A	A	A	A	A
154	1	5	4	-	-	-	-	-	-	-	-	-	-	-		C	C	C	C	C	-	-	-	-	-	-	C	C	C	C	C	C	C
155	1	5	5	A	A	A	A	A	A	A	A	A	A	A		A	A	A	A	A	A	A	A	A	A	A	A	A	A	A	A	A	A
156	1	5	6	C	C	C	C	C	C	C	C	C	C	C		C	C	C	C	C	C	C	C	C	C	C	C	C	C	C	C	C	C
157	1	5	7	A	A	A	A	A	A	A	A	A	A	A		A	A	A	A	A	A	A	A	A	A	A	A	A	A	A	A	A	A
158	1	5	8	A	A	A	A	A	A	A	A	A	A	A		A	A	A	A	A	A	A	A	A	A	A	A	A	A	A	A	A	A
159	1	5	9	A	A	A	A	A	A	A	A	A	A	A		A	A	A	A	A	A	A	A	A	A	A	A	A	A	A	A	A	A
160	1	6	0	A	A	A	A	A	A	A	A	A	A	A		A	A	A	A	A	A	A	A	A	A	A	A	A	A	A	A	A	A
161	1	6	1	G	G	G	G	G	G	G	G	G	G	G		G	G	G	G	G	G	G	G	G	G	G	G	G	G	G	G	G	G
162	1	6	2	C	C	C	C	C	C	C	C	C	C	C		C	C	C	C	C	C	C	C	C	C	C	C	C	C	C	C	C	C
163	1	6	3	A	A	A	A	A	A	A	A	A	A	A		A	A	A	A	A	A	A	A	A	A	A	A	A	A	A	A	A	A
164	1	6	4	A	A	A	A	A	A	A	A	A	A	A		A	A	A	A	A	A	A	A	A	A	A	A	A	A	A	A	A	A
165	1	6	5	A	A	A	A	A	A	A	A	A	A	A		A	A	A	A	A	A	A	A	A	A	A	A	A	A	A	A	A	A
166	1	6	6	A	A	A	A	A	A	A	A	A	A	A		A	A	A	A	A	A	A	A	A	A	A	A	A	A	A	A	A	A
167	1	6	7	A	A	A	A	A	A	A	A	A	A	A		A	A	A	A	A	A	A	A	A	A	A	A	A	A	A	A	A	A
168	1	6	8	T	T	T	T	T	T	T	T	T	T	T		T	T	T	T	T	T	T	T	T	T	T	T	T	T	T	T	T	T
169	1	6	9	G	G	G	G	G	G	G	G	G	G	G		G	G	G	G	G	G	G	G	G	G	G	G	G	G	G	G	G	G
170	1	7	0	T	T	T	T	T	T	T	T	T	T	T		T	T	T	T	T	T	T	T	T	T	T	T	T	T	T	T	T	T
171	1	7	1	C	C	C	C	C	C	C	C	C	C	C		C	C	C	C	C	C	C	C	C	C	C	C	C	C	C	C	C	C
172	1	7	2	C	C	C	C	C	C	C	C	C	C	C		C	C	C	C	C	C	C	C	C	C	C	C	C	C	C	C	C	C
173	1	7	3	G	G	G	G	G	G	G	G	G	G	G		G	G	G	G	G	G	G	G	G	G	G	G	G	G	G	G	G	G
174	1	7	4	T	T	T	T	T	T	T	T	T	T	T		T	T	T	T	T	T	T	T	T	T	T	T	T	T	T	T	T	T
175	1	7	5	T	T	T	T	T	T	T	T	T	T	T		T	T	T	T	T	T	T	T	T	T	T	T	T	T	T	T	T	T
176	1	7	6	T	T	T	T	T	T	T	T	T	T	T		T	T	T	T	T	T	T	T	T	T	T	T	T	T	T	T	T	T
177	1	7	7	A	A	A	A	A	A	A	A	A	A	A		A	A	A	A	A	A	A	A	A	A	A	A	A	A	A	A	A	A
178	1	7	8	T	T	T	T	T	T	T	T	T	T	T		T	T	T	T	T	T	T	T	T	T	T	T	T	T	T	T	T	T
179	1	7	9	A	A	A	A	A	A	A	A	A	A	A		A	A	A	A	A	A	A	A	A	A	A	A	A	A	A	A	A	A
180	1	8	0	C	C	C	C	C	C	C	C	C	C	C		C	C	C	C	C	C	C	C	C	C	C	C	C	C	C	C	C	C

				JQ730001.1 L. donovani isolate Trashiqang1	MW053322.1 L. donovani isolate 3	OM220625.1 L. donovani isolate MW23	OM220617.1 L. donovani isolate PK604	MK211306.1 L. donovani isolate TM-222 BD 2014 SB	MW053324.1 L. donovani isolate 5	MW053328.1 L. donovani isolate 9	CL18 (ON146457)	CL22 (ON146458)	CL25 (ON146459)	CL40 (ON146460)		KP691595.1 L. tropica isolate GSKH 1	OK338429.1 L. tropica isolate 356/TN/19	OK354361.1 L. tropica isolate 393/TN/19	KY963130.1 L. tropica isolate Pv76	KY963132.1 L. tropica isolate PvMBF	KX096623.1 L. infantum isolate 2013/MO13	MN422062.1 L. infantum isolate 35	MN503524.1 L. infantum isolate 37	MN503525.1 L. infantum isolate 42	MN503526.1 L. infantum isolate 29	MN503527.1 L. infantum isolate 23	AY573187.1 L. major strain MHOM/IR/00/PII2	MT023531.1 L. major voucher MK-MZ-DM4	KX821679.1 L. major strain MHOM/AT/16/AS16	CL17 (ON146453)	CL23 (ON146454)	CL29tissue (ON146455)	CL29culture (ON146456)
				donovani	donovani	donovani	donovani	donovani	donovani	donovani	donovani	donovani	donovani	donovani		tropica	tropica	tropica	tropica	tropica	infantum	infantum	infantum	infantum	infantum	infantum	major	major	major	major	major	major	major
181	1	8	1	-	-	-	-	-	-	-	-	-	-	-		-	-	-	-	-	-	-	-	-	-	-	A	A	A	A	A	A	A
182	1	8	2	A	A	A	A	A	A	A	A	A	A	A		-	-	-	-	-	A	A	A	A	A	A	A	A	A	A	A	A	A
183	1	8	3	A	A	A	A	A	A	A	A	A	A	A		-	-	-	-	-	A	A	A	A	A	A	A	A	A	A	A	A	A
184	1	8	4	A	A	A	A	A	A	A	A	A	A	A		-	-	-	-	-	A	A	A	A	A	A	A	A	A	A	A	A	A
185	1	8	5	A	A	A	A	A	A	A	A	A	A	A		A	A	A	A	A	A	A	A	A	A	A	A	A	A	A	A	A	A
186	1	8	6	A	A	A	A	A	A	A	A	A	A	A		A	A	A	A	A	A	A	A	A	A	A	A	A	A	A	A	A	A
187	1	8	7	A	A	A	A	A	A	A	A	A	A	A		A	A	A	A	A	A	A	A	A	A	A	A	A	A	A	A	A	A
188	1	8	8	T	T	T	T	T	T	T	T	T	T	T		T	T	T	T	T	T	T	T	T	T	T	T	T	T	T	T	T	T
189	1	8	9	A	A	A	A	A	A	A	A	A	A	A		A	A	A	A	A	A	A	A	A	A	A	A	A	A	A	A	A	A
190	1	9	0	T	T	T	T	T	T	T	T	T	T	T		T	T	T	T	T	T	T	T	T	T	T	G	G	G	G	G	G	G
191	1	9	1	A	A	A	A	A	A	A	A	A	A	A		A	A	A	A	A	A	A	A	A	A	A	A	A	A	A	A	A	A
192	1	9	2	C	C	C	C	C	C	C	C	C	C	C		C	C	C	C	C	C	C	C	C	C	C	C	C	C	C	C	C	C
193	1	9	3	G	G	G	G	G	G	G	G	G	G	G		G	G	G	G	G	G	G	G	G	G	G	G	G	G	G	G	G	G
194	1	9	4	G	G	G	G	G	G	G	G	G	G	G		G	G	G	G	G	G	G	G	G	G	G	G	G	G	G	G	G	G
195	1	9	5	C	C	C	C	C	C	C	C	C	C	C		C	C	C	C	C	C	C	C	C	C	C	C	C	C	C	C	C	C
196	1	9	6	G	G	G	G	G	G	G	G	G	G	G		G	G	G	G	G	G	G	G	G	G	G	G	G	G	G	G	G	G
197	1	9	7	T	T	T	T	T	T	T	T	T	T	T		T	T	T	T	T	T	T	T	T	T	T	T	T	T	T	T	T	T
198	1	9	8	T	T	T	T	T	T	T	T	T	T	T		T	T	T	T	T	T	T	T	T	T	T	T	T	T	T	T	T	T
199	1	9	9	T	T	T	T	T	T	T	T	T	T	T		T	T	T	T	T	T	T	T	T	T	T	T	T	T	T	T	T	T
200	2	0	0	C	C	C	C	C	C	C	C	C	C	C		C	C	C	C	C	C	C	C	C	C	C	C	C	C	C	C	C	C
201	2	0	1	G	G	G	G	G	G	G	G	G	G	G		G	G	G	G	G	G	G	G	G	G	G	G	G	G	G	G	G	G
202	2	0	2	G	G	G	G	G	G	G	G	G	G	G		G	G	G	G	G	G	G	G	G	G	G	G	G	G	G	G	G	G
203	2	0	3	T	T	T	T	T	T	T	T	T	T	T		T	T	T	T	T	T	T	T	T	T	T	T	T	T	T	T	T	T
204	2	0	4	T	T	T	T	T	T	T	T	T	T	T		T	T	T	T	T	T	T	T	T	T	T	T	T	T	T	T	T	T
205	2	0	5	T	T	T	T	T	T	T	T	T	T	T		T	T	T	T	T	T	T	T	T	T	T	T	T	T	T	T	T	T
206	2	0	6	-	-	-	-	-	-	-	-	-	-	-		T	T	T	T	T	-	-	-	-	-	-	-	-	-	-	-	-	-
207	2	0	7	-	-	-	-	-	-	-	-	-	-	-		G	G	G	G	G	-	-	-	-	-	-	-	-	-	-	-	-	-
208	2	0	8	T	T	T	T	T	T	T	T	T	T	T		T	T	T	T	T	T	T	T	T	T	T	T	T	T	T	T	T	T
209	2	0	9	T	T	T	T	T	T	T	T	T	T	T		T	T	T	T	T	T	T	T	T	T	T	T	T	T	T	T	T	T
210	2	1	0	G	G	G	G	G	G	G	G	G	G	G		G	G	G	G	G	G	G	G	G	G	G	G	G	G	G	G	G	G
211	2	1	1	G	G	G	G	G	G	G	G	G	G	G		G	G	G	G	G	G	G	G	G	G	G	G	G	G	G	G	G	G
212	2	1	2	C	C	C	C	C	C	C	C	C	C	C		C	C	C	C	C	C	C	C	C	C	C	C	C	C	C	C	C	C
213	2	1	3	G	G	G	G	G	G	G	G	G	G	G		-	-	-	-	-	G	G	G	G	G	G	G	G	G	G	G	G	G
214	2	1	4	G	G	G	G	G	G	G	G	G	G	G		-	-	-	-	-	G	G	G	G	G	G	G	G	G	G	G	G	G
215	2	1	5	-	-	-	-	-	-	-	-	-	-	-		-	-	-	-	-	-	-	-	-	-	-	G	G	G	G	G	G	G
216	2	1	6	-	-	-	-	-	-	-	-	-	-	-		-	-	-	-	-	-	-	-	-	-	-	A	A	A	A	A	A	A
217	2	1	7	-	-	-	-	-	-	-	-	-	-	-		-	-	-	-	-	-	-	-	-	-	-	G	G	G	G	G	G	G
218	2	1	8	-	-	-	-	-	-	-	-	-	-	-		-	-	-	-	-	-	-	-	-	-	-	G	G	G	G	G	G	G
219	2	1	9	-	-	-	-	-	-	-	-	-	-	-		-	-	-	-	-	-	-	-	-	-	-	G	G	G	G	G	G	G
220	2	2	0	-	-	-	-	-	-	-	-	-	-	-		-	-	-	-	-	-	-	-	-	-	-	A	A	A	A	A	A	A
221	2	2	1	-	-	-	-	-	-	-	-	-	-	-		-	-	-	-	-	-	-	-	-	-	-	G	G	G	G	G	G	G
222	2	2	2	-	-	-	-	-	-	-	-	-	-	-		-	-	-	-	-	-	-	-	-	-	-	A	A	A	A	A	A	A
223	2	2	3	-	-	-	-	-	-	-	-	-	-	-		-	-	-	-	-	-	-	-	-	-	-	G	G	G	G	G	G	G
224	2	2	4	-	-	-	-	-	-	-	-	-	-	-		-	-	-	-	-	-	-	-	-	-	-	A	A	A	A	A	A	A
225	2	2	5	-	-	-	-	-	-	-	-	-	-	-		-	-	-	-	-	-	-	-	-	-	-	G	G	G	G	G	G	G
226	2	2	6	-	-	-	-	-	-	-	-	-	-	-		-	-	-	-	-	-	-	-	-	-	-	A	A	A	A	A	A	A
227	2	2	7	G	G	G	G	G	G	G	G	G	G	G		G	G	G	G	G	G	G	G	G	G	G	G	G	G	G	G	G	G
228	2	2	8	G	G	G	G	G	G	G	G	G	G	G		G	G	G	G	G	G	G	G	G	G	G	G	G	G	G	G	G	G
229	2	2	9	T	T	T	T	T	T	T	T	T	T	T		G	G	G	G	G	T	T	T	T	T	T	G	G	G	G	G	G	G
230	2	3	0	G	G	G	G	G	G	G	G	G	G	G		G	G	G	G	G	G	G	G	G	G	G	G	G	G	G	G	G	G
231	2	3	1	G	G	G	G	G	G	G	G	G	G	G		G	G	G	G	G	G	G	G	G	G	G	G	G	G	G	G	G	G
232	2	3	2	G	G	G	G	G	G	G	G	G	G	G		G	G	G	G	G	G	G	G	G	G	G	G	G	G	G	G	G	G
233	2	3	3	T	T	T	T	T	T	T	T	T	T	T		T	T	T	T	T	T	T	T	T	T	T	T	T	T	T	T	T	T
234	2	3	4	G	G	G	G	G	G	G	G	G	G	G		G	G	G	G	G	G	G	G	G	G	G	G	G	G	G	G	G	G
235	2	3	5	C	C	C	C	C	C	C	C	C	C	C		C	C	C	C	C	C	C	C	C	C	C	C	C	C	C	C	C	C
236	2	3	6	G	G	G	G	G	G	G	G	G	G	G		G	G	G	G	G	G	G	G	G	G	G	G	G	G	G	G	G	G
237	2	3	7	T	T	T	T	T	T	T	T	T	T	T		T	T	T	T	T	T	T	T	T	T	T	T	T	T	T	T	T	T
238	2	3	8	-	-	-	-	-	-	-	-	-	-	-		G	G	G	G	G	-	-	-	-	-	-	G	G	G	G	G	G	G
239	2	3	9	-	-	-	-	-	-	-	-	-	-	-		T	T	T	T	T	-	-	-	-	-	-	C	C	C	C	C	C	C
240	2	4	0	G	G	G	G	G	G	G	G	G	G	G		G	G	G	G	G	G	G	G	G	G	G	G	G	G	G	G	G	G

				JQ730001.1 L. donovani isolate Trashigang1	MW053322.1 L. donovani	OM220625.1 L. donovani isolate MW23	OM220617.1 L. donovani isolate PK604	MK211306.1 L. donovani isolate TM-222 BD 2014 SB	MW053324.1 L. donovani	MW053328.1 L. donovani	CL18 (ON146457)	CL22 (ON146458)	CL25 (ON146459)	CL40 (ON146460)		KP691595.1 L tropica isolate GSKH1	OK338429.1 L tropica isolate 356/TN/19	OK354361.1 L tropica isolate 393/TN/19	KY963130.1 L tropica isolate Pv76	KY963132.1 L tropica isolate PvMBF	KX096623.1 L. infantum isolate 2013/M013	MN422062.1 L. infantum isolate 35	MN503524.1 L. infantum isolate 37	MN503525.1 L. infantum isolate 42	MN503526.1 L. infantum isolate 29	MN503527.1 L. infantum isolate 23	AY573187.1 L. major strain MHOM/IR/00/PII2	MT023531.1 L. major voucher MK-MZ-DM4	KX821679.1 L. major strain MHOM/AT/16/AS16	CL17 (ON146453)	CL23 (ON146454)	CL29tissue (ON146455)	CL29culture (ON146456)
				donovani	donovani	donovani	donovani	donovani	donovani	donovani	donovani	donovani	donovani	donovani		tropica	tropica	tropica	tropica	tropica	infantum	infantum	infantum	infantum	infantum	infantum	major	major	major	major	major	major	major
241	2	4	1	T	T	T	T	T	T	T	T	T	T	T		T	T	T	T	T	T	T	T	T	T	T	C	C	C	C	C	C	C
242	2	4	2	G	G	G	G	G	G	G	G	G	G	G		G	G	G	G	G	G	G	G	G	G	G	G	G	G	G	G	G	G
243	2	4	3	T	T	T	T	T	T	T	T	T	T	T		T	T	T	T	T	T	T	T	T	T	T	T	T	T	T	T	T	T
244	2	4	4	G	G	G	G	G	G	G	G	G	G	G		G	G	G	G	G	G	G	G	G	G	G	G	G	G	G	G	G	G
245	2	4	5	G	G	G	G	G	G	G	G	G	G	G		G	G	G	G	G	G	G	G	G	G	G	G	G	G	G	G	G	G
246	2	4	6	A	A	A	A	A	A	A	A	A	A	A		A	A	A	A	A	A	A	A	A	A	A	A	A	A	A	A	A	A
247	2	4	7	T	T	T	T	T	T	T	T	T	T	T		T	T	T	T	T	T	T	T	T	T	T	T	T	T	T	T	T	T
248	2	4	8	A	A	A	A	A	A	A	A	A	A	A		A	A	A	A	A	A	A	A	A	A	A	A	A	A	A	A	A	A
249	2	4	9	A	A	A	A	A	A	A	A	A	A	A		A	A	A	A	A	A	A	A	A	A	A	A	A	A	A	A	A	A
250	2	5	0	C	C	C	C	C	C	C	C	C	C	C		C	C	C	C	C	C	C	C	C	C	C	C	C	C	C	C	C	C
251	2	5	1	G	G	G	G	G	G	G	G	G	G	G		G	G	G	G	G	G	G	G	G	G	G	G	G	G	G	G	G	G
252	2	5	2	G	G	G	G	G	G	G	G	G	G	G		G	G	G	G	G	G	G	G	G	G	G	G	G	G	G	G	G	G
253	2	5	3	C	C	C	C	C	C	C	C	C	C	C		C	C	C	C	C	C	C	C	C	C	C	C	C	C	C	C	C	C
254	2	5	4	T	T	T	T	T	T	T	T	T	T	T		T	T	T	T	T	T	T	T	T	T	T	T	T	T	T	T	T	T
255	2	5	5	C	C	C	C	C	C	C	C	C	C	C		C	C	C	C	C	C	C	C	C	C	C	C	C	C	C	C	C	C
256	2	5	6	A	A	A	A	A	A	A	A	A	A	A		A	A	A	A	A	A	A	A	A	A	A	A	A	A	A	A	A	A
257	2	5	7	C	C	C	C	C	C	C	C	C	C	C		C	C	C	C	C	C	C	C	C	C	C	C	C	C	C	C	C	C
258	2	5	8	A	A	A	A	A	A	A	A	A	A	A		A	A	A	A	A	A	A	A	A	A	A	A	A	A	A	A	A	A
259	2	5	9	T	T	T	T	T	T	T	T	T	T	T		T	T	T	T	T	T	T	T	T	T	T	T	T	T	T	T	T	T
260	2	6	0	A	A	A	A	A	A	A	A	A	A	A		A	A	A	A	A	A	A	A	A	A	A	A	A	A	A	A	A	A
261	2	6	1	A	A	A	A	A	A	A	A	A	A	A		A	A	A	A	A	A	A	A	A	A	A	A	A	A	A	A	A	A
262	2	6	2	C	C	C	C	C	C	C	C	C	C	C		C	C	C	C	C	C	C	C	C	C	C	C	C	C	C	C	C	C
263	2	6	3	G	G	G	G	G	G	G	G	G	G	G		G	G	G	G	G	G	G	G	G	G	G	G	G	G	G	G	G	G
264	2	6	4	T	T	T	T	T	T	T	T	T	T	T		T	T	T	T	T	T	T	T	T	T	T	T	T	T	T	T	T	T
265	2	6	5	G	G	G	G	G	G	G	G	G	G	G		G	G	G	G	G	G	G	G	G	G	G	G	G	G	G	G	G	G
266	2	6	6	T	T	T	T	T	T	T	T	T	T	T		T	T	T	T	T	T	T	T	T	T	T	T	T	T	T	T	T	T
267	2	6	7	C	C	C	C	C	C	C	C	C	C	C		C	C	C	C	C	C	C	C	C	C	C	C	C	C	C	C	C	C
268	2	6	8	G	G	G	G	G	G	G	G	G	G	G		G	G	G	G	G	G	G	G	G	G	G	G	G	G	G	G	G	G
269	2	6	9	C	C	C	C	C	C	C	C	C	C	C		C	C	C	C	C	C	C	C	C	C	C	C	C	C	C	C	C	C
270	2	7	0	G	G	G	G	G	G	G	G	G	G	G		G	G	G	G	G	G	G	G	G	G	G	G	G	G	G	G	G	G
271	2	7	1	A	A	A	A	A	A	A	A	A	A	A		A	A	A	A	A	A	A	A	A	A	A	A	A	A	A	A	A	A
272	2	7	2	T	T	T	T	T	T	T	T	T	T	T		T	T	T	T	T	T	T	T	T	T	T	T	T	T	T	T	T	T
273	2	7	3	G	G	G	G	G	G	G	G	G	G	G		G	G	G	G	G	G	G	G	G	G	G	G	G	G	G	G	G	G
274	2	7	4	G	G	G	G	G	G	G	G	G	G	G		G	G	G	G	G	G	G	G	G	G	G	G	G	G	G	G	G	G
275	2	7	5	A	A	A	A	A	A	A	A	A	A	A		A	A	A	A	A	A	A	A	A	A	A	A	A	A	A	A	A	A
276	2	7	6	T	T	T	T	T	T	T	T	T	T	T		T	T	T	T	T	T	T	T	T	T	T	T	T	T	T	T	T	T
277	2	7	7	G	G	G	G	G	G	G	G	G	G	G		G	G	G	G	G	G	G	G	G	G	G	G	G	G	G	G	G	G
278	2	7	8	A	A	A	A	A	A	A	A	A	A	A		A	A	A	A	A	A	A	A	A	A	A	A	A	A	A	A	A	A
279	2	7	9	C	C	C	C	C	C	C	C	C	C	C		C	C	C	C	C	C	C	C	C	C	C	C	C	C	C	C	C	C
280	2	8	0	T	T	T	T	T	T	T	T	T	T	T		T	T	T	T	T	T	T	T	T	T	T	T	T	T	T	T	T	T
281	2	8	1	T	T	T	T	T	T	T	T	T	T	T		T	T	T	T	T	T	T	T	T	T	T	T	T	T	T	T	T	T
282	2	8	2	G	G	G	G	G	G	G	G	G	G	G		G	G	G	G	G	G	G	G	G	G	G	G	G	G	G	G	G	G
283	2	8	3	G	G	G	G	G	G	G	G	G	G	G		G	G	G	G	G	G	G	G	G	G	G	G	G	G	G	G	G	G
284	2	8	4	C	C	C	C	C	C	C	C	C	C	C		C	C	C	C	C	C	C	C	C	C	C	C	C	C	C	C	C	C
285	2	8	5	T	T	T	T	T	T	T	T	T	T	T		T	T	T	T	T	T	T	T	T	T	T	T	T	T	T	T	T	T
286	2	8	6	T	T	T	T	T	T	T	T	T	T	T		T	T	T	T	T	T	T	T	T	T	T	T	T	T	T	T	T	T
287	2	8	7	C	C	C	C	C	C	C	C	C	C	C		C	C	C	C	C	C	C	C	C	C	C	C	C	C	C	C	C	C
288	2	8	8	C	C	C	C	C	C	C	C	C	C	C		C	C	C	C	C	C	C	C	C	C	C	C	C	C	C	C	C	C
289	2	8	9	T	T	T	T	T	T	T	T	T	T	T		T	T	T	T	T	T	T	T	T	T	T	T	T	T	T	T	T	T
290	2	9	0	A	A	A	A	A	A	A	A	A	A	A		A	A	A	A	A	A	A	A	A	A	A	A	A	A	A	A	A	A
291	2	9	1	T	T	T	T	T	T	T	T	T	T	T		T	T	T	T	T	T	T	T	T	T	T	T	T	T	T	T	T	T
292	2	9	2	T	T	T	T	T	T	T	T	T	T	T		T	T	T	T	T	T	T	T	T	T	T	T	T	T	T	T	T	T
293	2	9	3	T	T	T	T	T	T	T	T	T	T	T		T	T	T	T	T	T	T	T	T	T	T	T	T	T	T	T	T	T
294	2	9	4	C	C	C	C	C	C	C	C	C	C	C		C	C	C	C	C	C	C	C	C	C	C	C	C	C	C	C	C	C
295	2	9	5	G	G	G	G	G	G	G	G	G	G	G		G	G	G	G	G	G	G	G	G	G	G	G	G	G	G	G	G	G
296	2	9	6	T	T	T	T	T	T	T	T	T	T	T		T	T	T	T	T	T	T	T	T	T	T	T	T	T	T	T	T	T
297	2	9	7	T	T	T	T	T	T	T	T	T	T	T		T	T	T	T	T	T	T	T	T	T	T	T	T	T	T	T	T	T
298	2	9	8	G	G	G	G	G	G	G	G	G	G	G		G	G	G	G	G	G	G	G	G	G	G	G	G	G	G	G	G	G
299	2	9	9	A	A	A	A	A	A	A	A	A	A	A		A	A	A	A	A	A	A	A	A	A	A	A	A	A	A	A	A	A
300	3	0	0	A	A	A	A	A	A	A	A	A	A	A		A	A	A	A	A	A	A	A	A	A	A	A	A	A	A	A	A	A
301	3	0	1	G	G	G	G	G	G	G	R	R	R	R		G	G	G	G	G	G	G	G	G	G	G	G	G	G	G	G	G	G
302	3	0	2	A	A	A	A	A	A	A	A	A	A	A		A	A	A	A	A	A	A	A	A	A	A	A	A	A	A	A	A	A
303	3	0	3	A	A	A	A	A	A	A	A	A	A	A		A	A	A	A	A	A	A	A	A	A	A	A	A	A	A	A	A	A
304	3	0	4	C	C	C	C	C	C	C	C	C	C	C		C	C	C	C	C	C	C	C	C	C	C	C	C	C	C	C	C	C
305	3	0	5	G	G	G	G	G	G	G	G	G	G	G		G	G	G	G	G	G	G	G	G	G	G	G	G	G	G	G	G	G
306	3	0	6	C	C	C	C	C	C	C	C	C	C	C		C	C	C	C	C	C	C	C	C	C	C	C	C	C	C	C	C	C
307	3	0	7	A	A	A	A	A	A	A	A	A	A	A		A	A	A	A	A	A	A	A	A	A	A	A	A	A	A	A	A	A
308	3	0	8	G	G	G	G	G	G	G	G	G	G	G		G	G	G	G	G	G	G	G	G	G	G	G	G	G	G	G	G	G
309	3	0	9	T	T	T	T	T	T	T	T	T	T	T		T	T	T	T	T	T	T	T	T	T	T	T	T	T	T	T	T	T
310	3	1	0	A	A	A	A	A	A	A	A	A	A	A		A	A	A	A	A	A	A	A	A	A	A	A	A	A	A	A	A	A
311	3	1	1	A	A	A	A	A	A	A	A	A	A	A		A	A	A	A	A	A	A	A	A	A	A	A	A	A	A	A	A	A
312	3	1	2	A	A	A	A	A	A	A	A	A	A	A		A	A	A	A	A	A	A	A	A	A	A	A	A	A	A	A	A	A

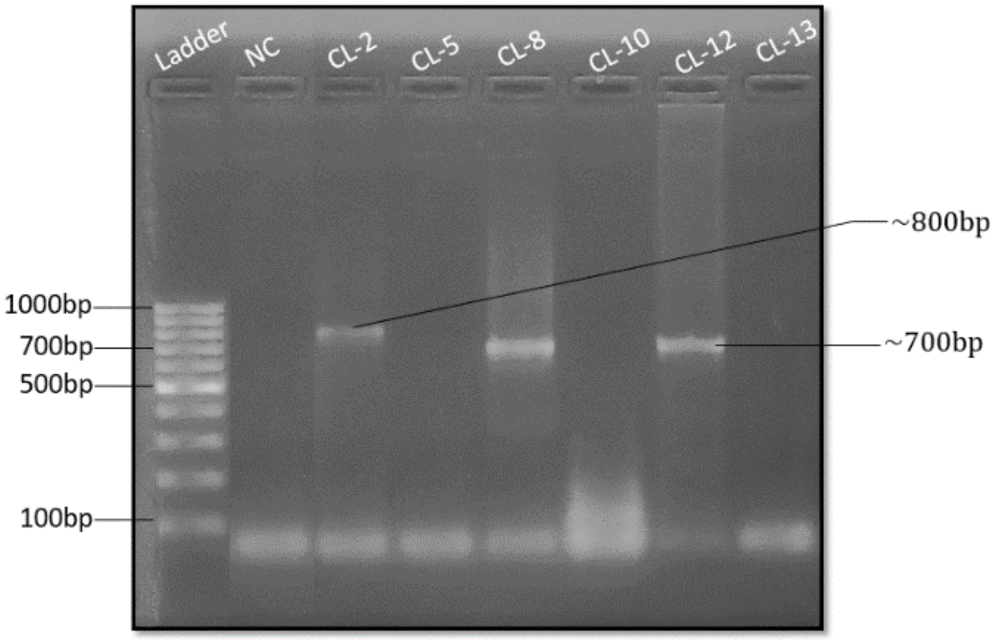

Supplementary Figure S-V: Minicircle kDNA PCR-1 assay of clinical samples. DNA isolated from the skin biopsies run for gel electrophoresis on 1.5% agarose gel and analysis. Lane1- 100 bp ladder, Lane 2- Negative control (NC), Lane 3-8 cutaneous leishmaniasis (CL) samples, Lane-3 (~800bp), Lane- 5&7, (~700bp), Lane 4,6,8- Not visualized

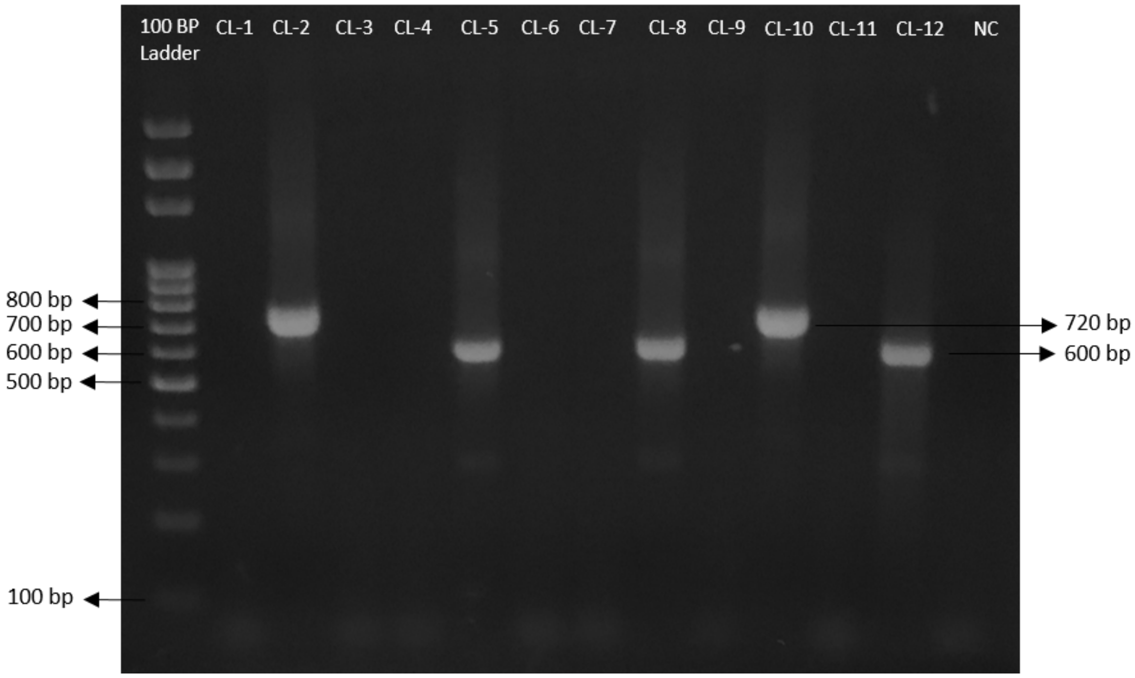

Supplementary Figure S-VI: Minicircle kDNA PCR-2 assay of clinical samples. PCR 1 amplicons of DNA isolated from the skin biopsies run for gel electrophoresis on 1.5% agarose gel and analysis. Lane1- 100 bp ladder, Lane 2- Lane 13, amplified product of skin biopsies of cutaneous leishmaniasis patients. NC, Negative control (No DNA). Lane 6, Lane 9 and Lane 13 (~600 bp of *L. major*), L3 and L11 (~720 bp *L. donovani*)

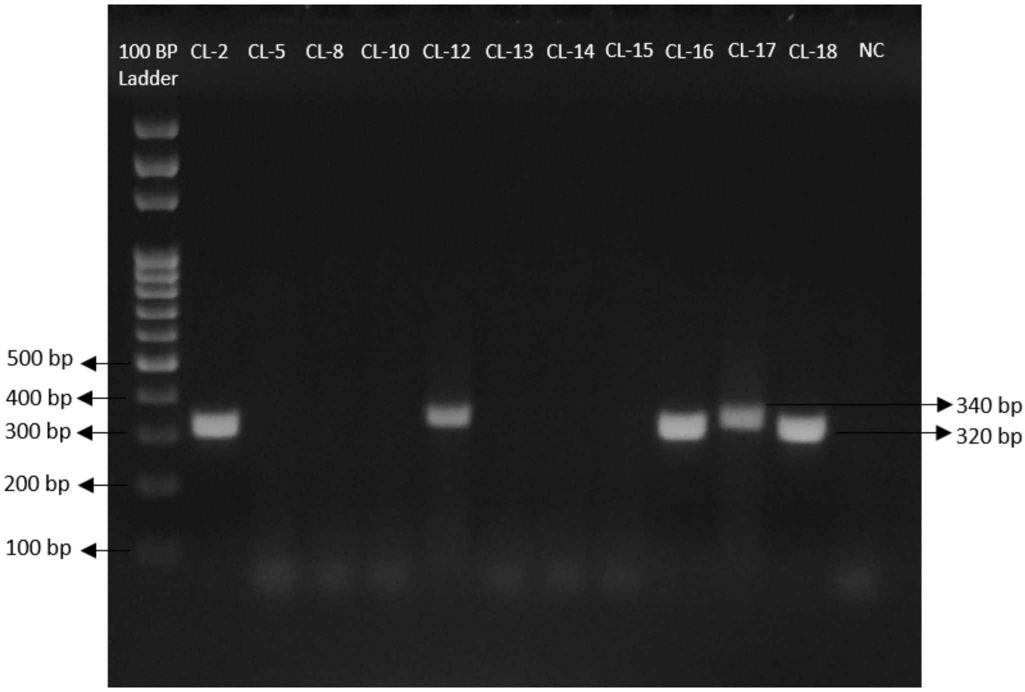

Supplementary Figure S-VII: ITS1 PCR assay of clinical samples. DNA isolated from the skin biopsies from the lesion was amplified and analyzed on 1.5% agarose gel. Lane1- 100 bp ladder, Lane2-Lane12- amplified product of skin biopsies of cutaneous leishmaniasis patients. NC, Negative control. Lane2, Lane10 and Lane12 (~320 bp of *L. donovani*), Lane6 and Lane11 (~340 bp of *L. major*).

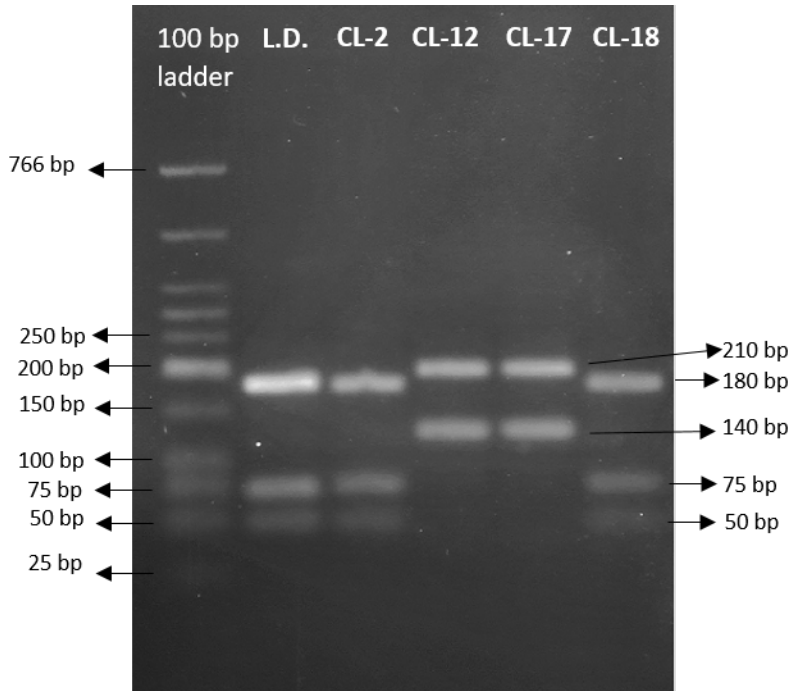

Supplementary Figure S-VIII: ITS1 RFLP assay of ITS1 PCR positive samples. Lane1- 100 bp ladder, Lane2, *L. donovani* (LEM 138), Lane3 and Lane6 (180bp, 75bp and 50bp of *L. donovani*), Lane4 and Lane5 (210 bp and 140 bp of *L. major*).
